# Quantitative videomicroscopy reveals latent control of cell-pair rotations *in vivo*

**DOI:** 10.1242/dev.200975

**Published:** 2023-05-03

**Authors:** Eva L. Kozak, Jerónimo R. Miranda-Rodríguez, Augusto Borges, Kai Dierkes, Alessandro Mineo, Filipe Pinto-Teixeira, Oriol Viader-Llargués, Jérôme Solon, Osvaldo Chara, Hernán López-Schier

**Affiliations:** ^1^ Unit Sensory Biology and Organogenesis, Helmholtz Zentrum München, Munich D-85764, Germany; ^2^ Systems Biology Group (SysBio), Institute of Physics of Liquids and Biological Systems (IFLySIB), National Scientific and Technical Research Council (CONICET), University of La Plata, La Plata B1900BTE, Argentina; ^3^ Graduate School of Quantitative Biosciences, Ludwig Maximilian University, Munich 81377, Germany; ^4^ Centre for Genomic Regulation, Barcelona 08003, Spain; ^5^ Instituto Biofisika, Basque Excellence Research Centre, Leioa 48940, Spain; ^6^ School of Biosciences, University of Nottingham, Sutton Bonington Campus, Nottingham LE12 5RD, UK; ^7^ Instituto de Tecnología, Universidad Argentina de la Empresa, Buenos Aires C1073AAO, Argentina; ^8^ Division of Science, New York University Abu Dhabi, Saadiyat Island 129188, United Arab Emirates

**Keywords:** Multicellular rotations, Patterning, Regeneration, Symmetry breaking, Zebrafish

## Abstract

Collective cell rotations are widely used during animal organogenesis. Theoretical and *in vitro* studies have conceptualized rotating cells as identical rigid-point objects that stochastically break symmetry to move monotonously and perpetually within an inert environment. However, it is unclear whether this notion can be extrapolated to a natural context, where rotations are ephemeral and heterogeneous cellular cohorts interact with an active epithelium. In zebrafish neuromasts, nascent sibling hair cells invert positions by rotating ≤180° around their geometric center after acquiring different identities via Notch1a-mediated asymmetric repression of Emx2. Here, we show that this multicellular rotation is a three-phasic movement that progresses via coherent homotypic coupling and heterotypic junction remodeling. We found no correlation between rotations and epithelium-wide cellular flow or anisotropic resistive forces. Moreover, the Notch/Emx2 status of the cell dyad does not determine asymmetric interactions with the surrounding epithelium. Aided by computer modeling, we suggest that initial stochastic inhomogeneities generate a metastable state that poises cells to move and spontaneous intercellular coordination of the resulting instabilities enables persistently directional rotations, whereas Notch1a-determined symmetry breaking buffers rotational noise.

## Teaser

**Highlighted Article:** Relative intercellular asymmetry is a crucial factor underlying the robustness of persistently directional collective cell rotations *in vivo*.

## INTRODUCTION

Collective cell movement is widespread during the formation and regeneration of organs (Lecaudey et al., 2008; Norden and Lecaudey, 2019; Dalle Nogare et al., 2020; Founounou et al., 2021; Alhashem et al., 2022; Hartmann and Mayor, 2022). This multicellular behavior is controlled at three levels: the onset, the progression and the conclusion. Each level generates the initial conditions for the next, and transition periods synchronize multiple sub- and supra-cellular processes to generate a predictable outcome (Gómez-Gálvez et al., 2021; Fredberg, 2022). At the extremes, the coordination of such processes may be deterministic and guided globally, or stochastic and canalized by local interaction and feedback between cells (Collinet and Lecuit, 2021; Hartmann and Mayor, 2022; Wibowo et al., 2011; Mirkovic et al., 2012; Tanner et al., 2012; Wang et al., 2013; Horne-Badovinac, 2014; Cetera et al., 2018; Hirata et al., 2018).

**Fig. 1.**
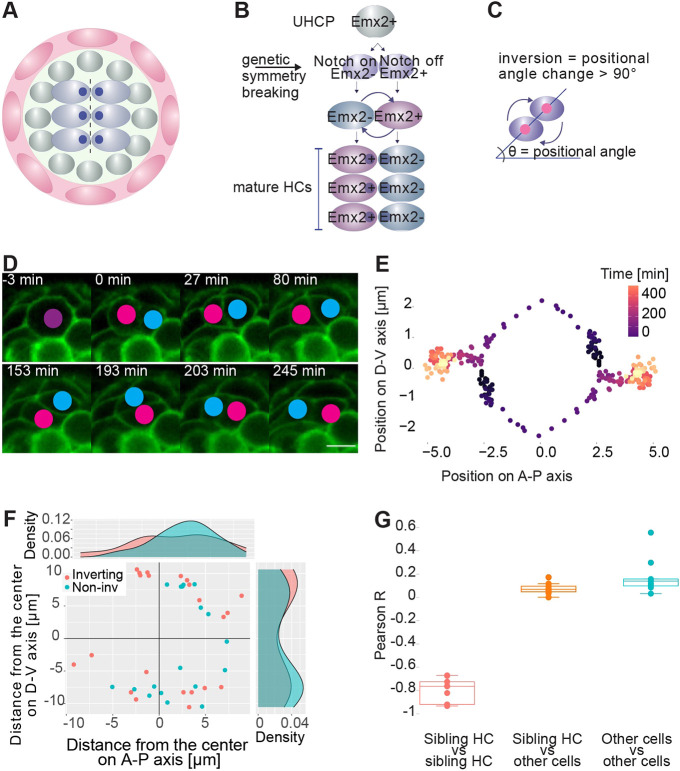
**Inversions are local movements of nascent sibling hair cells.** (A) Scheme of a neuromast, depicting an outer ring of mantle cells (red), internal supporting cells (gray) and central hair cells (light blue) with their axis of planar polarity (dark blue dots). Dashed line indicates the midline of the organ. (B) Scheme of hair-cell development. Unipotent progenitors (UHCP) divide into two hair cells. Sibling hair cells undergo positional inversion to place Notch-on/Emx2(−) and Notch-on/Emx2(+) cells on opposite sides of the epithelium. (C) The inversion is an angular movement of at least 90°. (D) Selected frames from a time-lapse movie of cell-pair inversion in a wild-type neuromast expressing cldnb:EGFP and myo6b:GFP. The timings are relative to the mitotic division that generates the hair-cell pair. Scale bar: 5 µm. (E) One exemplary hair-cell dyad during an inversion. The position of each cell during the inversion is depicted relative to the centroid of the pair. Time is color-coded from dark violet to yellow, where 0 is the time immediately after cell division and 400 is the upper limit of the inversion. (F) Position of the hair-cell progenitor at the time of its mitotic division. The color of the dots indicates whether the resulting hair-cell pair inverts (red) or not (blue). The center was defined as the position of all pre-existing hair cells in the neuromast. Side panels show the density of points along the dorsoventral (D-V) and anteroposterior (A-P) axes of the epithelium. It shows 22 inverting and 18 non-inverting hair-cell pairs from different neuromasts in 22 specimens. (G) Boxplots showing the Pearson correlation coefficient for the movement of cells in the neuromast along the A-P axis. Each point represents the cells during a rotation. Box plots show median values (middle bars) and first (Q1) to third (Q3) interquartile ranges (boxes); upper whisker is either 1.5× the interquartile range or the maximum value (whichever is the smallest) and lower whisker is either 1.5× the interquartile range or the minimum value (whichever is the biggest). For each neuromast, the movement was compared between the rotating hair cells (HC); the rotating hair cells and all other cells; and between all other cells. *n*=9 independent neuromasts from *N*=9 different larvae.

Here, we focus on a minimal model of collective cell rotations involving the positional inversion of just two cells, which was first described in neuromasts of the zebrafish lateral line (Wibowo et al., 2011). Neuromasts display largely invariant size and pattern. They consist of a radial-symmetric epithelium containing mechanosensory hair cells in the center, and two types of non-sensory supporting cells forming two outward concentric rings (Fig. 1A) (Wada and Kawakami, 2015). Hair cells are also polarized along a single axis across the apical face of the epithelium (López-Schier and Hudspeth, 2006). Hair cells undergo continuous renewal without modifying the architecture of the organ (Cruz et al., 2015; Pinto-Teixeira et al., 2015; Peloggia et al., 2021). During turnover, hair cells are produced sequentially, in pairs or dyads, from the mitotic division of facultative unipotent progenitors (UHCP) that originate from internal supporting cells (López-Schier and Hudspeth, 2006; Ma et al., 2008; Cruz et al., 2015; Denans et al., 2019; Thomas and Raible, 2019; Hardy et al., 2021; Baek et al., 2022). Local lateral-inhibitory signaling via Notch1a breaks the initial symmetry in nascent sibling hair cells by repressing the transcription factor Emx2 in one of them (Jacobo et al., 2019; Kozak et al., 2020). The cell that activates the Notch1a receptor (Notch-on) loses Emx2 expression, whereas its sibling (Notch-off) maintains it. Although this symmetry-breaking process is deterministic in that it always results in one of the siblings losing Emx2 expression, it is also stochastic because it is unpredictable which cell will do so. Concurrently with this step, around half of the hair-cell pairs rotate once around their geometric center (Fig. 1B-D and Movie 1) (Wibowo et al., 2011; Mirkovic et al., 2012).

Notably, cell-pair rotations *in vivo* resemble the angular movement of mammalian cells *in vitro*, which is driven by three co-occurring processes: intrinsic cell motility and stochastic symmetry breaking (to initiate movement), strong intercellular adhesion (enabling dynamic coupling between cells for persistent directionality) and spatial confinement (so that cells cannot translocate) (Brangwynne et al., 2000; Huang et al., 2005; Camley et al., 2014; Leong, 2013; Li and Sun, 2014; Huang, 2016). Also, cell-pair rotations *in vitro* happen nearly always, have no predictable duration or extent, and do not involve intrinsic differences between cells or interactions with a surrounding epithelium. They are monotonous, exhibiting symmetrical sinusoidal trajectories with almost invariable frequency and amplitude (Huang et al., 2005). Moreover, theoretical studies have strongly influenced our thinking about multicellular rotations (Huang et al., 2005; Camley et al., 2014; Li and Sun, 2014; Leong, 2013). Yet, the mechanistic overlap between rotating cells *ex vivo* and in a natural context remains unknown. Here, we combine videomicroscopy, experimental perturbations and modeling to quantitatively characterize cell-pair rotation *in vivo*, and reveal previously-overlooked features affecting rotational precision.

## RESULTS

### Cell-pair rotation *in vivo* is a discrete movement of nascent sibling hair cells

We began by acquiring a highly resolving dataset from intravital videomicroscopy of neuromasts in the posterior lateral line of larval zebrafish (Pinto-Teixeira et al., 2013). We used specimens expressing a combination of fluorescent transgenic markers to identify and visualize every neuromast cell (Haas and Gilmour, 2006; Kindt et al., 2012; López-Schier and Hudspeth, 2006; Steiner et al., 2014). To quantify rotations at high resolution, we first defined the angle between the axis connecting the center of each cell of nascent pairs and the horizontal axis of the neuromast, which invariably runs parallel to the anteroposterior axis of the animal's body (Fig. 1C). Sibling hair cells invert positions by moving in circular arcs around their geometric center (Fig. 1E). We used a strict definition of inversion as a rotation of at least 90° of the line connecting the center of both cells at the time of their birth. Angular movements lower than 90° were considered local rearrangements rather than inversions. We confirmed results from previous studies, that ∼50% of hair-cell dyads inverted, whereas the other half underwent transient rocking movements that did not result in a net positional exchange between the cells (Wibowo et al., 2011; Mirkovic et al., 2012; Ohta et al., 2020). We also found a similar frequency of inverting and stationary cell pairs in horizontal and vertical neuromasts (56.3% and 47.4%, respectively).

We did not see any significant bias from these rules when comparing inverting cell pairs at different positions along the orthogonal axes of the neuromast, suggesting that the localization of the cell-pair within the organ does not determine or tune rotations (Fig. 1F). Moreover, we never observed hair cells translocating across the tissue, indicating the inversions are a purely local collective movement. To directly test whether the rotation is an active process autonomous to the inverting cell pairs or otherwise driven by the action of neighboring cells, we assessed the movement of every cell across the entire epithelium (Fig. S1A). We segmented cellular boundaries from live imaging of neuromasts expressing a plasma-membrane targeted EGFP. Then, we quantified the displacement of each cell using particle tracking while keeping the center of the neuromast spatially fixed (Movie 2). This allowed us to compute the Pearson correlation coefficient R for the trajectories in nine independent datasets. We found that the rotation of hair-cell pairs is highly anticorrelated (R=−0.77), reflecting local translocation of cells. This was expected given that the trajectory of each hair cell is almost perfectly mirror-symmetric relative to the centroid of the cell pair (Fig. 1E). However, we found that epithelium-wide cellular movement is uncorrelated (R=0.14). This means that the movement of any one cell did not correlate with that of any other cell taken at random, indicating no coherent epithelium-wide cellular flow. Importantly, the movement of the rotating cell pairs is uncorrelated with the rest of the epithelium (R=0.07), indicating that rotations are not driven by any fixed anisotropic force (Fig. 1G). These data further reinforce the conclusion that cell-pair inversion is an autonomous active process of physically confined nascent hair cells.

### Cell-pair inversion is triphasic and characterized by temporally correlated strong homotypic contacts and coordinated heterotypic junctional remodeling

**Fig. 2.**
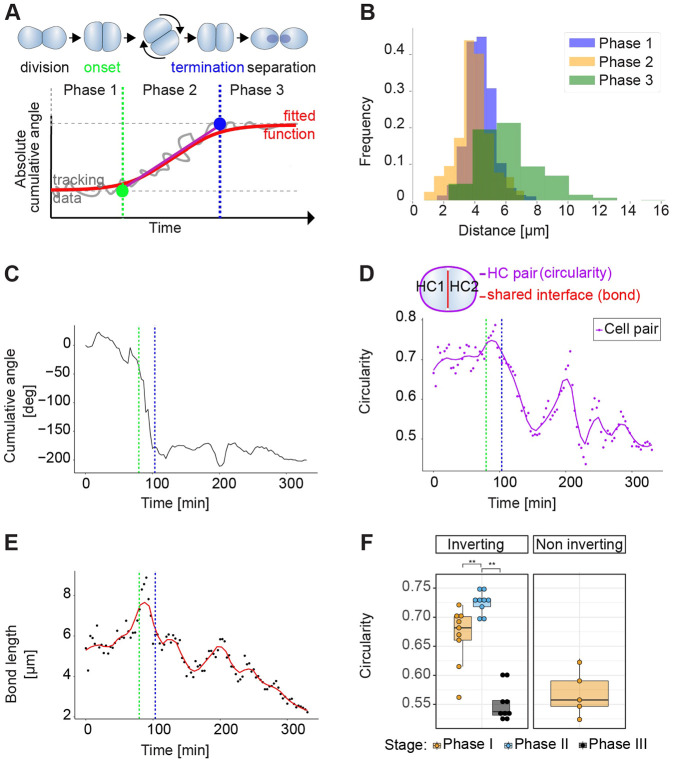
**Rotations are characterized by strong homotypic cell-cell interactions.** (A) Scheme of an inversion (top) and fitting a four-parameter logistic function to the empiric data of the absolute cumulative angles (bottom), revealing the three phases of the inversion process. Green dot represents the transition between Phases 1 and 2, and the blue dot between Phases 2 and 3. These transitions are called onset and termination, respectively. (B) Distance between sibling hair cells (HC) during the three phases. Sibling cells are closest during Phase 2. (C) Time-resolved cumulative angular change (top) for a representative cell pair from Time 0. The two dashed vertical lines mark the beginning and end of the rotation. A negative angle change indicates a clockwise direction of rotation. (D) Circularity of the same cell pair, which, taken as a unit, reaches maximal circularity during Phase 2. The onset and termination of inversion are marked by dashed vertical lines. (E) Length of the junction between hair cell pairs, which is highest during Phase 2. (F) Comparison of mean circularity for inverting and non-inverting cell pairs during the three phases. Phase 2 is characterized by a significantly higher circularity than Phase 1, and the circularity drops dramatically in Phase 3. The circularity for non-inverting pairs is comparable to the Phase 3 of inverting pairs. Trivially, for non-inverting cells there is a single phase. **P<0.01, Wilcoxon rank sum test. Box plots show median values (middle bars) and first (Q1) to third (Q3) interquartile ranges (boxes); upper whisker is either 1.5× the interquartile range or the maximum value (whichever is the smallest) and lower whisker is either 1.5× the interquartile range or the minimum value (whichever is the biggest). Each point represents the cells during a rotation.

Evolving changes in cell shape as well as junctional interphase length and shape indicate the dynamics of forces acting upon cells. This includes intrinsic intra- and inter-cellular forces as well extrinsic forces from neighboring cells (Maître and Heisenberg, 2011; Yap et al., 2018; Lenne et al., 2021). Therefore, we decided to investigate the above morphological features during the rotations. To this end, we established a generalizable standard to benchmark this and future studies by continuously measuring the positional angle of rotating cell pairs and computing the absolute cumulative angle over time. Using a four-parameter logistic function that provided a good fit for the empirical data, we found that cell-pair rotations can be clearly split into three phases with unique characteristics. Phase 1 is the period between the birth of the hair-cell pair and the onset of rotation, Phase 2 is the time where the main angular movement occurs and Phase 3 follows the end of active rotation until the cell pair reaches its final position (Fig. 2A).

The three phases differ in several important ways. In rotating cell pairs, the distance between the center of each cell remains constant during Phase 1, drops significantly during Phase 2 and then increases again in Phase 3 (Fig. 2B). In Phase 1, the cumulative angle of movement stays close to 0. Phase 2 starts with a rapid change in the cumulative angle, ending within a maximal rotation of 180° at Phase 3. Fig. 2C shows one example of a rotating pair. During Phase 2, the circularity of the cell pair is low in Phase 1, high throughout Phase 2, and decreases sharply at Phase 3 (Fig. 2D), revealing that both cells deform in a correlated manner (Fig. S1B). Coincidently, there is a conspicuously fast change of the homotypic interphase (common junction) between the inverting cells, growing to a maximum during Phase 2, and shrinking again in Phase 3 (Fig. 2E). Also, the variation in circularity of the cell pair and of the length of their common junction correlate during all three phases (Fig. 2D-F; Fig. S1C). Importantly, none of these variations were observed in non-inverting pairs (Fig. 2F; Fig. S1D).

**Fig. 3.**
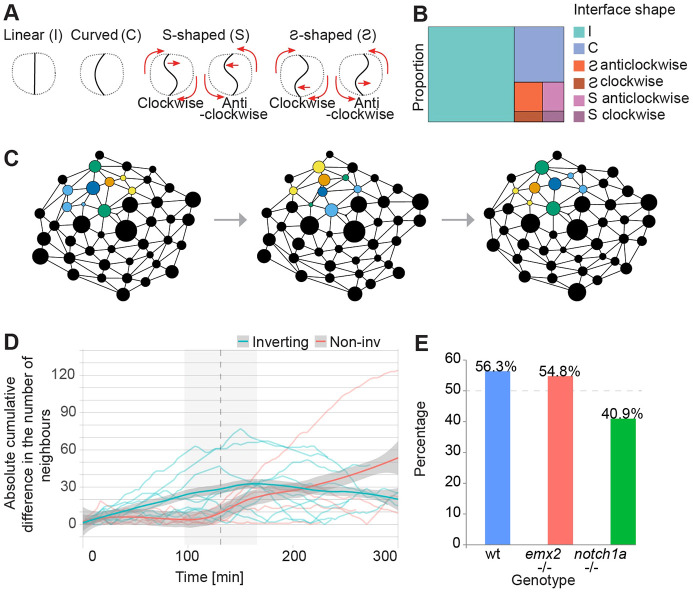
**Notch1a/Emx2 asymmetry is dispensable for cell-pair rotations.** (A) Illustration of the cell-cell interfaces, shapes of which are classified as curved (C), linear (I), S or Ƨ shaped. Because an Ƨ cannot be rotated into an S, a distinction is made of the rotation direction. (B) The frequency of distinct interface shapes during inversion. The most common shape is a straight interface (63.41%), followed by curved (21.31%) and then S-shaped curves (S: 6.57% and Ƨ: 8.7%). No shape consistently correlated with the chirality of rotation. (C) Scheme of the topological interactions between all epithelial cells during the inversion of a cell pair (representing one empirical example). Each circle is an individual cell. The area of each circle is proportional to the area of real cells from microscopy images. Straight lines (edges) represent a physical contact between any two cells. Cells are considered neighbors if there is an edge connecting them. A pair of hair cells is colored blue and orange. Neighboring cells are colored light blue if they connect only to the blue sibling in a given frame; yellow if they only connect to the other sibling; or green if they are connected to both. (D) Absolute cumulative difference in the number of neighbors for each cell of inverting (blue) and non-inverting (red) pairs. The difference of neighbors at a given time is the number of neighbors of Cell A minus the number of neighbors of Cell B. The cumulative difference at any given time T is the sum of the neighbor differences from time 1 to time *N*. Therefore, if there is no cell with a constantly higher number of neighbors over time, the cumulative difference remains close to zero. However, if either one of the cells constantly has more neighbors, over time the absolute cumulative difference will go up. Vertical dashed line and gray shaded areas mark the median and standard deviation of the Phase 2I for the inverting cells in this sample. Shown is the LOESS smoothing of inverting and non-inverting trajectories. (E) Fraction of hair-cell pairs that invert in wild type, *emx2* mutant and *notch1a* mutant larvae. *n* (number of cell pairs)=71 from wild type, 42 from *emx2* mutant, 22 from *notch1a* mutant larvae.

Rotating cell pairs *in vitro* display an invariant sigmoidal common junction (previously called ‘Yin-Yang shape’ in various *in vitro* and theoretical studies) (Brangwynne et al., 2000; Huang et al., 2005; Leong, 2013; Li and Sun, 2014). Notably, *in vitro*, the polarity of the sigmoid and the handedness of the rotations are always correlated, in that cell pairs rotate anti-clockwise upon S-shaped junctions and clockwise when junctions are Ƨ-shaped (Huang et al., 2005). This correlation has been explained by the effect of a front-end lamellipodium of one cell wrapping the trailing edge of the other cell (Brangwynne et al., 2000), which led to the conclusion that cells neither push, nor pull one another during rotations (Brangwynne et al., 2000). However, theoretical analyses concluded that the cells must employ rear pull during rotations (Camley et al., 2014; Leong, 2013). We decided to explore this provocative idea of rotating cells *in vivo* by classifying their homotypic interphase into four categories (Fig. 3A). Two of them are non-chiral: linear shape (I), and curved shape (C). Note that the C mirrored junction is non chiral because it can be rotated back: Ɔ⇔C. The remaining two are chiral (S and Ƨ) because they cannot be rotated into one other. By measuring interphase across focal planes in Phase 2, we found that in the majority of the cases they are symmetric and linear (I-shaped). Although we observed some S and Ƨ shapes, we found no correlation between their handedness and the direction of rotation (Fig. 3B; Movie 3).

### Notch1a and Emx2 differentially affect cell-pair rotations

The above results led us to hypothesize that sibling hair cells interact symmetrically with the adjacent epithelial cells. This is intriguing given that sibling hair cells are distinct from one another by virtue of their asymmetric Notch/Emx2 status (Jiang et al., 2017; Jacobo et al., 2019; Kozak et al., 2020; Erzberger et al., 2020; Kindt et al., 2021). Therefore, we searched for any consistent difference between sibling cells that may indicate that this molecular asymmetry is mechanistically linked to the rotation. To this end, we employed cellular- and temporal-resolved tracking to obtain a topological and dynamic representation of rotations (Movie 2). We extrapolated junctional dynamics by quantifying the difference in the number of neighbors of each hair cell at consecutive timepoints (Fig. 3C). Importantly, because the final position of each hair cell will reveal their Notch/Emx2 status, we could also retrospectively infer whether a cell is Notch-off/Emx2(+) or Notch-on/Emx2(−) before the onset of inversions in Phase 1. We hypothesized that, if neighbor exchange over time was symmetric, when one hair cell loses a neighbor it immediately recovers by gaining a new neighbor and vice versa. This is true even if the exchange of neighbors were not simultaneously experienced by both hair cells. Symmetry means that the difference between the number of neighboring cells that the rotating siblings will have is always zero. Any departure from zero indicates that the contact of Notch-off/Emx2(+) and Notch-on/Emx2(−) cells with neighboring epithelial cells is consistently different (that is, invariably asymmetric). Of note, cellular proliferation and death are negligible during the recording period, effectively ruling out neighbor gain or loss via changes in cell number. As expected, we found that the difference in the number of neighbors is zero for non-inverting cell pairs because they do not exchange neighbors (Fig. 3D). For inverting pairs, the accumulated sum diverged from zero from birth, indicating that one of the hair cells (that we call ‘popular’) consistently has more neighbors than its sibling (Fig. 3D), which is maintained throughout the rotation. Importantly, however, the identity of the popular cell could not be predicted from the dynamic data, indicating that the Notch/Emx2 status of a cell does not correlate with its popularity. These results led us to hypothesize that Notch/Emx2 asymmetry does not determine rotations, and that neither cell drives the movement. Importantly, this idea is in partial disagreement with the current model, which states that Emx2 is dispensable for rotations, whereas Notch1a is essential (Ohta et al., 2020; Erzberger et al., 2020). Therefore, we decided to directly test it using self-consistent experimental conditions, data acquisition and analysis. We recorded rotations in fish carrying homozygous loss-of-function mutations in Emx2 (Movie 4) or Notch1a (Movie 5). We first confirmed that rotations happen at normal frequency in *emx2* mutants (Fig. 3E) (Ohta et al., 2020). However, they are marginally less frequent in *notch1a* mutants (Fig. 3E). Put together, these data indicate that cell-pair rotation is characterized by co-occurring increase of hair-cell homotypic interactions and coherent heterotypic junction remodeling. Furthermore, the Notch-off/Emx2(+) and Notch-on/Emx2(−) cells participate in the rotation in an indistinguishable manner.

**Fig. 4.**
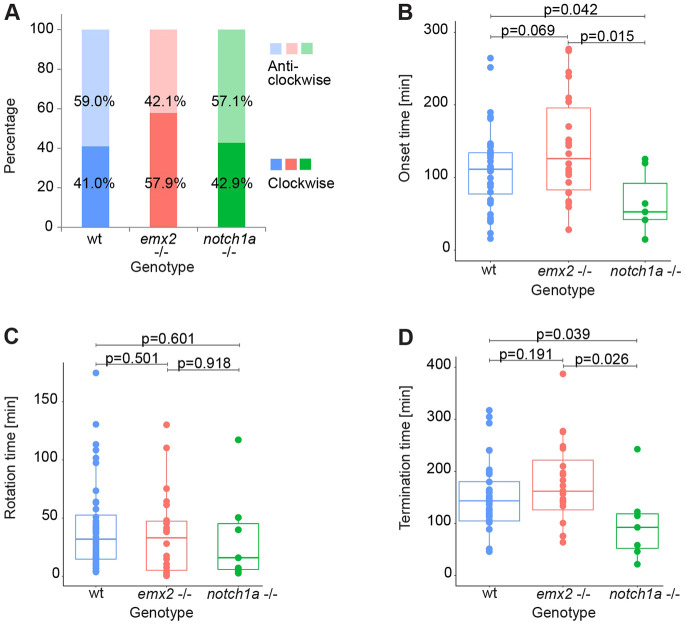
**Loss of Notch1a but not Emx2 impacts cell-pair rotations.** (A) Handedness of cell-pair inversions in wild-type, *emx2* and *notch1a* mutant larvae. (B) Comparison of the onset (duration of Phase 1) of inverting cell pairs from wild type, *emx2* and *notch1a* mutants. (C) Comparison of the rotation duration (Phase 2) of inverting cell-pairs from wild-type, *emx2* mutant and *notch1a* mutant larvae. (D) Comparison of the termination time (Start of Phase 3) of inverting cell pairs from wild-type, *emx2* mutant and *notch1a* mutant larvae. (A-D) *n* (number of cell pairs)=40 from wild-type, 23 from *emx2* mutant, 9 from *notch1a* mutant larvae. Statistics were calculated using an unpaired two-sided Student's *t*-test. Box plots show median values (middle bars) and first (Q1) to third (Q3) interquartile ranges (boxes); upper whisker is either 1.5× the interquartile range or the maximum value (whichever is the smallest) and lower whisker is either 1.5× the interquartile range or the minimum value (whichever is the biggest). Each point represents the cells during a rotation.

Intrigued by the previous results, we decided to perform a more detailed quantitative analysis of rotations across the three phases, comparing wild-type specimens with those carrying loss-of-function mutations in Emx2 and Notch1a (Fig. S1E-G). We found that the rotations in the wild type were approximately equally frequent in the clockwise and anti-clockwise directions. Similarly, *emx2* and *notch1a* mutants had negligible handedness bias (Fig. 4A). Following this, we fitted a sigmoid function to each rotating trajectory with respect to time to unbiasedly define the boundaries between the three phases (Fig. 2A; Fig. S1H-J). The onset of the active rotation phase (start of Phase 2) was typically ∼100 min after the birth of the hair cells, but with noticeable variability. We found that the start of Phase 2 was marginally delayed in *emx2* mutants, but significantly accelerated in *notch1a* mutants (Fig. 4B). The duration of Phase 2, however, did not differ between the three genotypes (Fig. 4C). As a consequence, cell-pairs in *notch1a* mutants arrived at Phase 3 earlier than in wild-type and *emx2* mutants (Fig. 4D).

### Effect of Notch/Emx2 asymmetry on the accuracy and precision of rotations

**Fig. 5.**
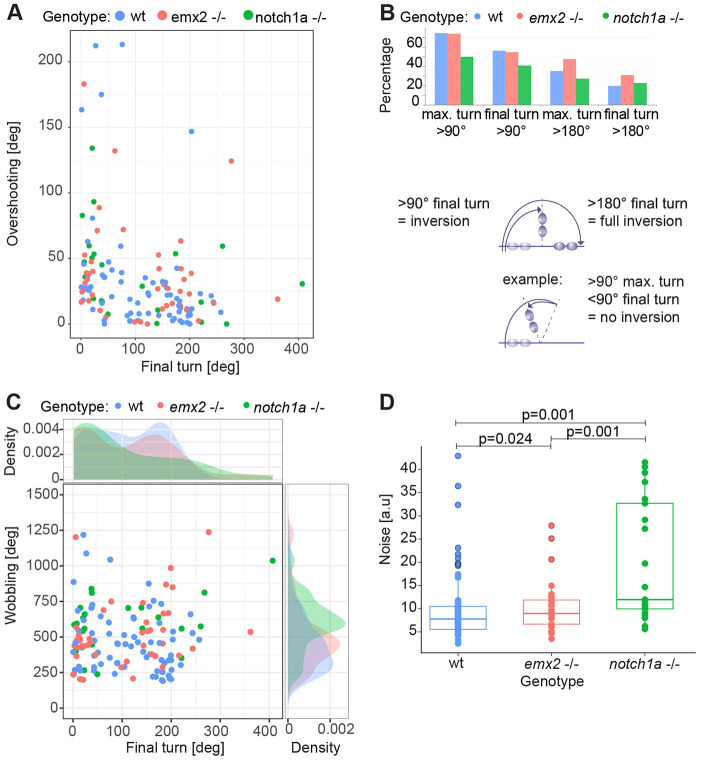
**Mutations in *notch1a* impact the precision of the inversion.** (A) Overshooting versus final turn in the rotations of cell pairs from wild type, *emx2* and *notch1a* mutants. We defined overshooting as the difference between the maximal turn and the final turn (absolute cumulative angle). (B) The maximal and final turns of >90° and >180° for cell pairs from wild-type, *emx2* mutant and *notch1a* mutant larvae. The sketches below illustrate the definition of categories with an example. (C) Comparison of cell pair wobbling and final turn. Wobbling was defined as the cumulative angle changes minus final turn. (D) Noise of rotating cell pairs was calculated as the arc length of each trajectory normalized by the shortest path from starting position to final position. Statistics were calculated using the Anderson-Darling test. (A-D) *n* (number of cell pairs)=71 from wild-type, 42 from *emx2* mutant, 22 from *notch1a* mutant larvae. Box plots show median values (middle bars) and first (Q1) to third (Q3) interquartile ranges (boxes); upper whisker is either 1.5× the interquartile range or the maximum value (whichever is the smallest) and lower whisker is either 1.5× the interquartile range or the minimum value (whichever is the biggest). Each point represents the cells during a rotation.

Tissue patterning is affected by both the accuracy and the precision of underlying dynamical processes (Mestek Boukhibar and Barkoulas, 2016). Importantly, although precision and accuracy are often used interchangeably, they represent non-trivial different parameters. A precise process displays a tight distribution of data points, regardless of the mean value. In other words, it has low variance. By contrast, an accurate process has a specific and consistent mean value, regardless of the actual variance of data points. It follows that loss of accuracy leads to an invariant scattering of data but with a significant deviation from mean values (akin to consistent but non-noisy changes), whereas a loss of precision will show higher scattering of data points but with non-significant changes in the mean values (akin to a noisier distribution). This distinction is important because it allows us to better compare wild-type, *emx2* and *notch1a* mutant specimens, to shed light on the aspects of the inversion process that are influenced by genetically-determined cell identity or cell-pair asymmetry. We first focused on the transition between Phases 2 and 3. Namely, whether cell pairs arrive at their final position in one single movement or whether they overshoot and then re-align to the main axis of the organ by either a single corrective movement or multiple approximating rocking movements. As a measure of overshoot, we subtracted the final turn (the final absolute cumulative angle) from the maximal turn (the maximal absolute cumulative angle) of the cell pair. We saw rare events of cell pairs performing a double inversion or reversals, arresting in their original position. These exceptional cases were equally frequent in all three genotypes (Fig. 5A,B). In most cases, the values of the maximal turn and the final turn were very close (Fig. 5B).

When measuring the whole angular movement, it became evident that rotations are not strictly monotonous because they include small-scale and recurrent swings, which we call ‘wobbling’. To quantify wobbling, we estimated the arc-length by summing the absolute angular changes. We found high wobbling in *emx2* mutants, and even higher in *notch1a* mutants (Fig. 5C). We further calculated noise as a related but unitless quantity of regularity, defined as the arc length of each trajectory normalized by the shortest path from starting to final position. The distributions for each experimental condition were statistically different (Fig. 5D), in that rotations were noisy in *emx2* mutants, and much noisier in *notch1a* mutants.

**Fig. 6.**
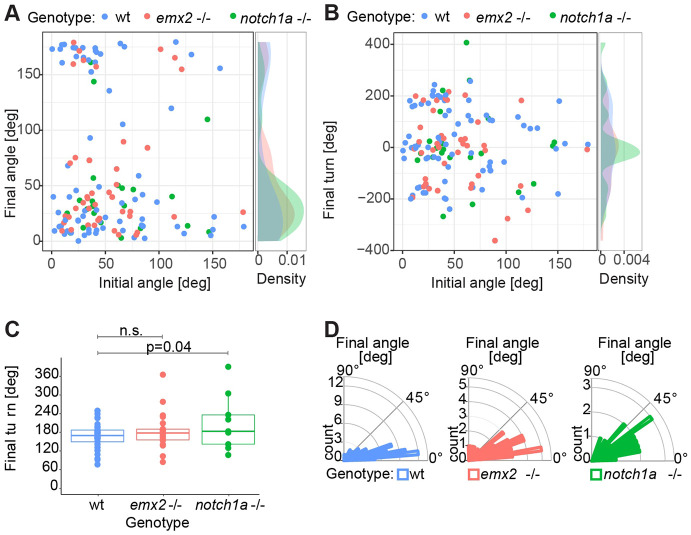
**Cell-pair asymmetry affects the precision and accuracy of Phase 3.** (A) Relationship between the final and initial angles. The initial angle is the positional angle of the cell pair immediately after the division. The final angle is where cells come to rest (regardless of whether they have rotated). Note that both angles were normalized to 0-180° (from 0-360°). (B) Relationship between the initial angle and the final turn of cell pairs of wild type, *emx2* mutant and *notch1a* mutants. Note the initial angle was normalized to 0-180° (from 0-360°). (C) Comparison of the final turn of cell pairs from wild type, *emx2* mutant and *notch1a* mutant larvae. Statistics were calculated using an unpaired two-sided Student's *t*-test. Box plots show median values (middle bars) and first (Q1) to third (Q3) interquartile ranges (boxes); upper whisker is either 1.5× the interquartile range or the maximum value (whichever is the smallest) and lower whisker is either 1.5× the interquartile range or the minimum value (whichever is the biggest). Each point represents the cells during a rotation. (D) Final angle of cell pairs from wild type, *emx2* and *notch1a* mutants. The difference in the distribution of final angles from wild type and the two mutants are statistically significant (*P*<0.05), Statistics were calculated using a two sample Kolmogorov–Smirnov test (*P*=0.004 for wild type and *emx2* mutant, and *P*=0.01 for wild type and *notch1a* mutant). (A-D) *n* (number of cell pairs)=71 from wild type, 42 from *emx2* mutant, 22 from *notch1a* mutants.

Next, we compared the initial and final positional angles (Phase 1 versus Phase 3). The initial angle corresponds to the position of the hair cells immediately after they are born. In the wild type, hair cells arise with a full spectrum of initial angles, but through rotations, the distribution of final angles becomes remarkably biphasic: either lower than 50° or higher than 150° (Fig. 6A). Note that a positional angle of either 0° or 180° means a perfect alignment with the anteroposterior axis of the neuromast. We speculated that the significant angular variability of Phase 1 (initiation) may be buffered through Phase 2 (active rotation) to reach a remarkably invariable alignment of the cells in Phase 3 (termination). The initial alignment of the cell pairs influences rotation handedness in order to undergo the lowest possible angular change (Fig. 6B). In other words, inverting cell pairs arising at 0°will tend to rotate 180°, whereas cells arising, for example at 30°, will rotate 150° rather than 210° in the opposite direction. We found the average final turn of rotating cell pairs is indistinguishable in wild type and *emx2* mutants, but larger in *notch1a* mutants (Fig. 6C). However, the final angle distributions in both mutants were significantly different from the wild type (Fig. 6D).

### A computer model of the inversion suggests that intercellular asymmetry simultaneously underlies rotational and positional precision

**Fig. 7.**
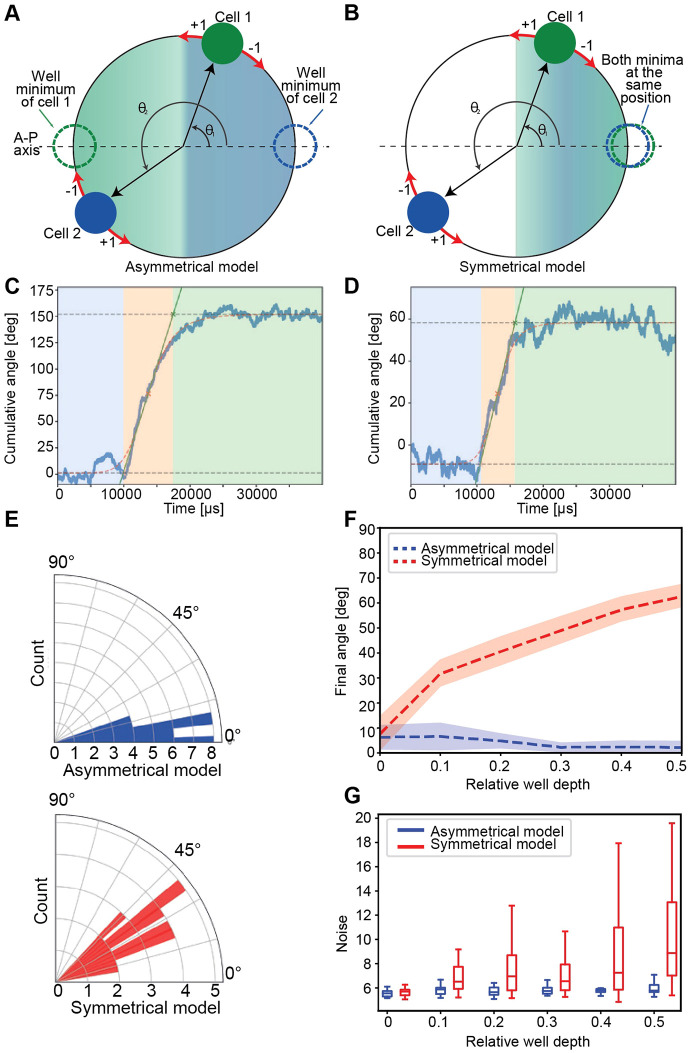
**Computational modeling of cell-pair inversion.** (A,B) Sketch of the two-cell computational model. Each cell freely rotates around a circle of a fixed radius, with its angle with respect to the *x*-axis (representing the A-P axis) as the degree of freedom for each cell. The arrows in each cell indicate the direction as +1 or −1 for anti-clockwise and clockwise movement, respectively. Each cell is attracted to one and only one Gaussian well. The depth of each well determines the strength with which its corresponding cell is attracted. The depth of one well can be different from the other. (A) In the asymmetrical model both cells have their attractive wells on opposite sides of the A-P axis. (B) In the symmetrical model both attractive wells lie on one side of the system, leading to a competition. (C,D) Two typical trajectories of the inverting pair in cumulative angle, as defined in Fig. 2C, for the asymmetrical and symmetrical model, respectively. The background colors indicate the phases of the process: blue (Phase 1), orange (Phase 2), green (Phase 3). (E) Distribution of final angles predicted by both models. Well depths are 0 and 50 (relative well depth=0) for the asymmetric model, and 50 and 20 (relative well depth=0.4) for the asymmetric model. (F,G) The final angle (F) and the noise (G) predicted by the asymmetric (symmetric) model are robust (sensitive) against the relative well depth. The depth of the reference well was fixed at 50 in these simulations. Asymmetric and symmetric models are represented in blue and red, respectively. Box plots show median values (middle bars) and first (Q1) to third (Q3) interquartile ranges (boxes); upper whisker is either 1.5× the interquartile range or the maximum value (whichever is the smallest) and lower whisker is either 1.5× the interquartile range or the minimum value (whichever is the biggest).

Our understanding of the mechanism governing the robustness of cell-pair inversions *in vitro* has enormously benefited from accompanying the experimental studies with solid theory. However, a theoretical framework of inversions *in vivo* has not yet been established. To remedy this shortcoming, we decided to develop a naïve computational model of inversions *in vivo.* We emphasized the rotational wobbling during Phase 2 and also the termination that corresponds to Phase 3. The reason behind this choice is that these events represent the main dynamic process of the inversion and are the ones experiencing the most significant deviations between the three genotypes analyzed in this study. First, we simulated the cell dyad as two particles that can freely rotate within a single plane about an orthogonal axis (Fig. 7A,B). This is appropriate because there is no evidence of anything preventing rotations once they start, and we hardly ever witnessed any off-place inversion. Of note, it is equivalent to having the rotational angle with respect to the *x*-axis as the only degree of freedom. Moreover, the *x*-axis was set to coincide with the anatomical anteroposterior axis of the neuromast. Because experimental data show that the final angle of wild-type cells falls within a narrow distribution (Fig. 6D), we reasoned that certain locations are strongly preferred. Therefore, we introduced attractive potentials into the model. Specifically, each cell is affected by an attractive potential modeled as a Gaussian well at a certain position in the circle, the depth of which represents the strength of the attraction. As our results indicate that only the inverting cell pair has a coordinated movement during the inversion process, we assumed that the role of the neighboring cells is to only confine the cell pair, with no active participation. Apart from the attraction wells, each cell of the pair interacts with each other through a soft sphere repulsion term, with an effective radius such that the cells are permanently in contact with one another, representing an effective spatial exclusion.

First, we tested a model in which each cell has its respective Gaussian well on the opposite sides of its location at the start of the inversion (Fig. 7A). Effectively, this means that a cell that appears in the anterior pole will have its minima in the posterior pole and vice versa. As each cell has its corresponding attractor in the opposite side of the anteroposterior (A-P) axis, we call this model asymmetrical. We found that the rotation that we modeled *in silico* qualitatively matches the empirical data from the wild type, in that the cell pair performs a stochastic rotation until each cell reaches its respective well (Fig. 7C; Movie 6). Notably, we also observed that if only one of the cells has an attractive well, the resulting dynamics are indistinguishable from the case where both cells have an attractive well (Movie 7). Specifically, in this case, one of the cells will be directed towards the minima, driving the inversion, whereas the other cell will passively move due to the spatial exclusion defined above. Importantly, the Notch1a/Emx2 identity of the ‘driving’ cell is irrelevant. Our experimental data show that the cell-pair rotations take place also in *emx2* and *notch1a* mutants, in which the symmetry of Emx2 expression is not broken.

Accordingly, we also generated a symmetrical model, in which both cells have attractive wells on the same side of the circle and compete to arrive at it (Fig. 7B). In this model, the cells undergo rotation until they reach a compromise between their mutual exclusion and the attraction to the minimum of the wells (Fig. 7D; Movie 8). Trivially, the particular case in which one of the wells has a depth of zero matches the one-well situation (Movie 7). After proper parametrization (see Materials and Methods), we simulated our model and quantified the final angle achieved by the cell pairs and the noise of the angular trajectory, defined as the arc length of each trajectory normalized by the shortest path from starting to final position. We found that the asymmetrical model is in best agreement with the wild-type experimental data (Fig. 7E). Most importantly, we found that in the symmetrical model the final angle strongly depends on the relative depth between the wells, but not on the absolute strength of attraction. Thus, when one of the wells has half the depth of the other, the final angle has a deviation of ∼60° with respect to the *x*-axis (Fig. 7F). We also saw that noise in the asymmetrical model was consistently lower than in the symmetrical model (Fig. 7G). Symmetry in the cell attractors results in more variability in the final angle (Fig. 7E,F), as well as higher wobbling (noisier dynamics) (Fig. 7C versus D, and Fig. 7G).

## DISCUSSION

**Fig. 8.**
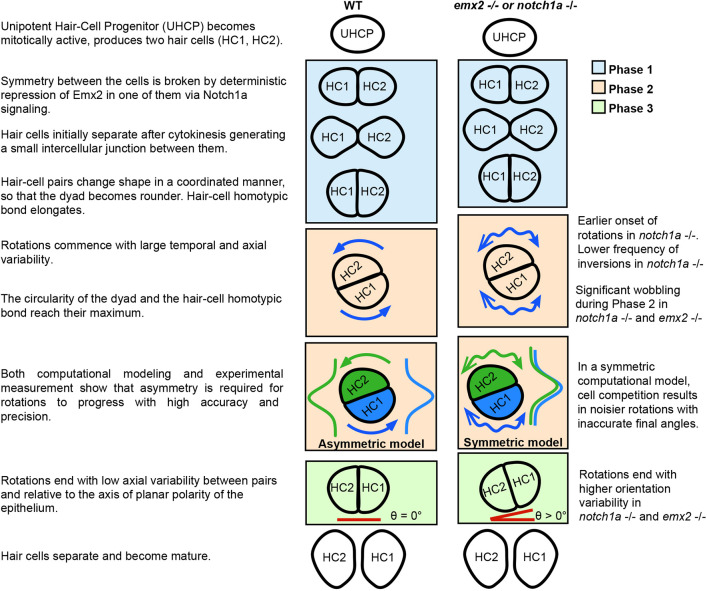
**A model of cell-pair rotation *in vivo*.** Overview of cell-pair inversion process summarizing the key elements of the inversions in wild type on the left, and stating major differences occurring in the *notch1a*^−/−^ and *emx2*^−/−^ larvae on the right. The precision of the angular movement *in vivo* approaches that of cells *in vitro*. Phase 1 starts immediately after division of the UHCP, is characterized by a coordinated shape of the cells and expansion of the homotypic bond. Phase 2 marks the maximum of circularity of the dyad, the length of the homotypic bond and of angular velocity. Mutant rotations are characterized by significant wobbling, denoted by double-headed arrows. A computational model recapitulates this dynamic difference just by assuming that the minima of energy potentials are either symmetric or asymmetric (green and blue curves represent the Gaussian wells to which minima the respective cells are attracted in our computational model). In Phase 3 the angle between the cells and the A-P axis (blue lines) is 0 with high accuracy and precision but it is misaligned in mutants. The symmetric and asymmetric models can also explain these differences.

Much of our understanding of multicellular rotations derives from experimental and theoretical studies of cells *in vitro* (Brangwynne et al., 2000; Huang et al., 2005; Tseng et al., 2012; Leong, 2013; Li and Sun, 2014; Camley et al., 2014; Segerer et al., 2015; Camley and Rappel, 2017; Brückner et al., 2021). Huang and colleagues proposed three essential conditions for cell-cohort rotation *in vitro*: (1) cells must be in a confined space; (2) cells should have a long-persistence time of intrinsic motility; (3) the cell dyad must be coupled by intercellular adhesion (Huang et al., 2005). Leong used interphase morphology and rotation chirality to introduce a particle-dynamics model to explain why dynamic coupling (the third condition) is essential for rotations (Leong, 2013). Along the same line, Camley and colleagues introduced a mean-field model to identify conditions under which cells would initiate a rotation (Camley et al., 2014). They proposed a confined system where each cell has a polarity defined by the evolution of a chemical signal. Notwithstanding these insightful theoretical milestones, the generality and relevance of their conclusions to cells rotating in their natural context has remained unknown. In this study, we fill this gap by focusing on a minimal model of collective cell movement *in vivo* involving the coherent rotation of two cells. Combining experiment, quantitative videomicroscopy and computer simulation, we establish the first model underlying the emergence and coherence of cell-pair rotations *in vivo* (Fig. 8).

Previous studies have suggested that Notch1a-mediated symmetry breaking via Emx2 is necessary for cell-pair inversions *in vivo* (Erzberger et al., 2020). Yet, independent work that forced symmetrical expression of the Notch1a target Emx2 showed marginal non-significant effects on rotations (Ohta et al., 2020). We interpreted this disagreement as suggesting that either genetically-determined cell-pair asymmetry via Notch1a/Emx2 and cell-pair inversions are epiphenomena, or that Notch1a controls rotations independently of its only known transcriptional target in neuromasts (Jacobo et al., 2019; Kozak et al., 2020). To address this discrepancy, we used a novel approach to quantitatively analyze the inversion process. We confirmed the predicted dispensability of Emx2 but, unexpectedly, found that ∼40% of hair-cell pairs inverted in specimens lacking Notch1a. We further demonstrated that neither Emx2 or Notch1a activity, nor Notch1a/Emx2 asymmetry between sibling cells, are essential for cell-pair rotations *in vivo*. These results allow us to consider various possibilities to explain the discrepancy of previous conclusions. First, neomorphic or gain-of-function mutant alleles in the genes under study may produce an atypical function that affects rotations (Guichard et al., 2002; Langdon et al., 2006), which may have led to the erroneous conclusion that Notch1a activity is essential for rotations. Second, passenger mutations are not uncommon across the genome of the zebrafish strains used in nearly every laboratory. Because previous studies used a single Notch1a mutant allele, the molecular profile of which remains unknown, and no rescue experiments were reported, this possibility cannot be overlooked (Erzberger et al., 2020). To solve these issues, we have combined unambiguous high-resolution quantitative determination of inversions, comprehensive statistical tests and two independently-generated *notch1a* mutant alleles, the molecular lesion of which has been well characterized, and from which we obtained an indistinguishable phenotype (Kozak et al., 2020). We found marginal statistical differences in rotation frequency between all three genotypes. Our results are unlikely to result from partial penetrance of the two *notch1a* mutant alleles that we have used because both had a very strong effect on other previously well-characterized phenotypes: neuromast epithelial bipolarity and somitogenesis (Kozak et al., 2020). Unexpectedly, however, we found that the loss of Notch1a produces noisier rotations without changes in mean values. This suggests that neither Notch1a activity nor Notch1a-mediated asymmetry impact the active or resistive forces that underlie rotations, and reveals that both Notch(on) and Notch(off) hair cells participate equally in the movement. In addition, although rotating cells consistently interact asymmetrically with the surrounding epithelium, their Notch/Emx2 status does not correlate with this asymmetry. Finally, the loss of Emx2 or Notch1a did not affect the speed of the rotation. Therefore, we conclude that Notch1a is not essential for inversions, and that both cells participate equally in the angular movement. We envision that it is not the action of Notch1a itself, but instead that of Notch1a-mediated cell-pair asymmetry which impacts rotational precision by generating a slight but persistent bias in microscopic dynamics. This leads to an increase in macroscopic coordination, with the consequent reduction of dynamic noise. Importantly, these findings reveal unanticipated latent control of rotational precision *in vivo*.

Moreover, we investigated the extent to which cell-pair inversion *in vivo* is a result of non-autonomous forces. We found no correlation between cell-pair rotations and epithelium-wide cellular flow, indicating that there are no anisotropic resistive forces from the surrounding epithelium. Alternatively, if anisotropic forces exist, they are not stably oriented (Yap et al., 2018; Bodor et al., 2020). We found that Phase 1 is unpredictable and highly variable, and that the onset of Phase 2 is very fast, strongly suggesting that Phase 1 is marked by instability. The dynamics of Phase 2 have low variability across the arc described by the cell pair. Although wobbling is noticeable, the rotational movement is persistently directional. Inertia is unlikely to explain persistence given the extremely low Reynolds numbers of biological tissues (Hakim and Silberzan, 2017). Instead, we speculate that directionality is driven by a ‘leaky’ ratchet mechanism that allows persistence despite wobbling (Caballero et al., 2020). Under this scenario, persistently directional rotations will emerge by spontaneous self-generating reciprocity between cells in physical confinement. This is further supported by the observation that the interphase between inverting cells is symmetric and linear (I-shaped) in the majority of the cases. Although we did find some S and Ƨ shapes, their handedness did not correlate with the direction of the rotation. This suggests that rotating cells *in vivo* do not exert consistent pulling or pushing forces upon one another. Therefore, rotating cell pairs in neuromasts may represent a vertebrate example of ‘contact following’, a mechanism that has been put forward to explain the coherent motion of *Dictyostelium* cells when they form circular rotating cohorts (Umeda and Inouye, 2002).

The molecular mechanism governing the extent (discontinuity) of the rotation (Phase 3) remains enigmatic. However, we also used a naïve computational model to advance on this question. We generated two models, called symmetrical and asymmetrical. Both models include a Gaussian well of minimal energy on the opposite sides of the location of each cell of the dyad at the start of the inversion process. However, in the asymmetrical model each cell has its corresponding attractor in the opposite side of the A-P axis of the neuromast. In the symmetrical model, both cells have attractive wells on the same side of the neuromast and compete to arrive at it. The symmetrical model best explains the experimental results of *emx2* and *notch1a* mutants, by assuming a different relative affinity for each set of potentials. By contrast, the asymmetrical model better recapitulates the robustness and final positions of the wild-type scenario. Hence, our theoretical framework suggests that the relative asymmetry of the cell attractors is the crucial element that underlies the robustness of the inversion process *in vivo*. Moreover, by testing models that recapitulate empirical data, we suggest that rotational movement will cease once the two-cell system reaches a low energy state (higher stability). Therefore, movement ceases when a ‘potential well’ or local minimum of potential energy exists, towards which the system will invariably and inevitably converge. This idea also explains another outstanding question: why do half of the cell dyads never rotate? Our model suggests that this occurs because a stable state takes hold before the coordination of local instabilities that leads to rotation can take place. Importantly, this argument would imply that the loss of Notch1a may not necessarily accelerate Phase 1 as we stated above, but simply prevent the late-onset rotations from taking place, coincidently skewing the onset towards lower values and decreasing the frequency of inversions.

We conclude that dyads of genetically equivalent cells can rotate if they are in a metastable state during which they experience persistent instabilities that enable them to move. A co-occurring spontaneous coordination of unbiased cellular motion would initiate rotations, whereas coherent homotypic interactions and heterotypic junction remodeling will enable directional persistence. Notch1a-mediated symmetry breaking between sibling cells acts as a stabilizer of the rotation. Our theoretical framework is important because it also suggests that relative asymmetry, rather than absolute attractiveness of potential wells, is the crucial element that underlies the robustness of the inversion process. This study exemplifies the power of combining high-resolution quantitative data with computational modeling to further understand the relationship between stochastic and deterministic processes underlying multicellular dynamics *in vivo*.

## MATERIALS AND METHODS

### Zebrafish lines and husbandry

Zebrafish larvae (*Danio rerio*) were kept under standard conditions at 28.5°C. The transgenic lines myo6b:β-actin-GFP (Kindt et al., 2012) and *Tg[−8.0cldnb:Lyn-EGFP]* (Haas and Gilmour, 2006), and the emx2LOF mutant line (Jiang et al., 2017) have been previously described. Notch1a CRISPR mutagenesis has been described in Kozak et al. (2020). We recovered two indel alleles hzm17 and hzm18. hzm17 is an indel disrupting the *notch1a* ORF at exon 16 and is kept in the myo6b:β-actin-GFP transgenic background. hzm18 is an indel causing the loss of part of exon 3 of *notch1a* and is kept in a myo6b:β-actin-GFP; claudnb:lyn-EGFP double transgenic background. Experiments were carried out either by crossing hzm17 to hzm18 or in-crossing hzm18.

### Imaging, image processing and data extraction

The time-lapse movies were generated using 2-3 days postfertilization MS222-anesthetized larvae mounted in 1% low-melting point agarose in a glass-bottom Petri dish. Up to five larvae were imaged simultaneously using a Zeiss custom-built inverted spinning-disc confocal microscope with a 63× water-immersion objective. For each stage position, stacks of 16-20 *z*-slices 1 µm apart were acquired every 200 s. In the videographs, newborn hair cells were identified retrospectively by playing the movies backwards from the time when hair cells can be unambiguously defined using validated transgenic markers (López-Schier and Hudspeth, 2006; Kindt et al., 2012). All 4D movies were processed using FIJI software (Schindelin et al., 2012). Stacks were centered by laying point regions of interest at timeframes of significant drift and then running the Manual Drift Correction plugin. Nine inverting pairs and five non inverting pairs were selected for image segmentation and were further registered for *z-*slice drifts using the plugin Correct 3D drift (Parslow et al., 2014).

### Comparison of cell-pair inversion

Nascent hair cells were manually tracked from the moment of division at a minimum 300 min with the MTrackJ plugin (Meijering et al., 2012). Cell tracking data (71, 42 and 22 cell pairs from wild type, *emx2* and *notch1a* knockouts, respectively) was imported into R (version 4.0.3), where all subsequent analysis was carried out. Individual cell positions were centered in pairwise fashion and observation time was limited to 500 min. For each observation time except the first, change in angle between cells relative to previous observation was calculated, positive angle denoting anti-clockwise rotation. Furthermore, for each cell pair and each observation time, cumulative angle (sum of angle changes) and absolute cumulative angle (named turn) were calculated. For each cell pair, starting angle and final angle were calculated as means of first ten or last ten observations, respectively. Final cumulative angle (final turn) and final cumulative absolute angle change were extracted as respective values at last observation. Critical angle defining planar cell inversion was set to 90°. If final cumulative angle change was higher than this critical value, the cells were considered to perform planar cell inversion. Two-sided binomial test was used to calculate statistics on the occurrence of cell-pair rotations in different genotypes relative to wild type. For the cell pairs that did undergo an inversion, a four parameter log-logistic curve was fitted to the cumulative angle using python ‘scipy.optimize’ library. The form of the logistic used was:

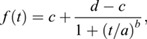
where *t* is the time, and *a*, *b*, *c* and *d* are the parameters to fit. Here, *a* is the time of the logistic midpoint, *b* is the steepness, *c* marks the low asymptote and the high asymptote.

Start and end times of inversion (*Ic* and *Id*) were calculated as the points where the tangent line through the inflection point and the low and high asymptote intersect, respectively:

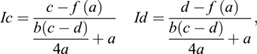
where *f*(*a*) is the fitted logistic function described above, evaluated at time *a*.

The noise was defined as the quotient between the real path traveled by the cell and the shortest path from start to end position according to:

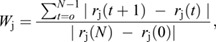
where *r*_j_ (*t*) is the position of cell j at time *t* in the *xy*-plane, and *N* is the number of frames in the experiment. Only the first 200 frames were used for this calculation. Those experiments where one of the two sibling cells moved less than 2 μm were not taken into account for the analysis. The resulting distributions for noise, as defined above, were analyzed by pairwise comparison using an Anderson-Darling test (AD) to see whether samples could be drawn from the same underlying distribution and with a Wilcoxon-Mann–Whitney statistic  (WMW)to test the alternative of the first distribution being stochastically less than the other. The *P*-values for the noise with both tests were: wild type versus *emx2*^−/−^, AD *P*=0.024, WMW *P*=0.022; wild type versus *notch1a*^−/−^, AD *P*<0.001, WMW *P*<0.0001; *emx2*^−/−^ versus *notch1a*^−/−^, AD *P*<0.001, WMW *P*<0.0001.

### Topological analysis of neuromast cells during hair cell inversion

After registration, one *z*-slice per time point was selected, taking 10 timepoints before division and at least 70 after. To segment membranes, we used the Autocontext workflow from ilastik v1.3.3 (Kreshuk and Zhang, 2019). As we used the double cldnb:lyn-EGFP; myo6b:β-actin-GFP transgenic, we trained in a first step, the probability of pixels to belong to one of four categories: membrane, cytoplasm, sub-membrane actin and background. In the second step, the probability of the four categories was used to train the algorithm to classify cell boundary pixels and all other pixels. The resulting probabilities were loaded in the multicut segmentation workflow to get a skeletonized segmentation of cells. These automated segmentations were loaded into Tissue analyzer (Aigouy et al., 2016) and manually corrected and semi automatically tracked. From the software Tissue analyzer, we exported two types of data: (1) cell tracking data containing *x* and *y* centroid position, cell area, perimeter (in pixels) and an ID identifying individual cells through time; (2) bond tracking data, indicating the identity of cells sharing a membrane segment, and the length of the membrane segment (in pixels). The data for each pair was fused to create a network dynamic object with the networkDynamic R package (v 0.10.1) containing information for nodes (cells) and edges (cell connections) through time as well as position, area, and perimeter.

Cumulative difference in number of neighbors is defined, for any given time *T*, as:

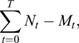
where *N*_*t*_ is the number of cell neighbors, at time *t*, of the cell that ultimately arrived at the anterior side irrespective of whether any rearrangement happened [the Notch-off/Emx2(+) cell] and *M*_*t*_ is the number of cell neighbors, at time *t*, of the cell that finishes the sequence on the posterior side irrespective of whether any rearrangement happened [the Notch-on/Emx2(−) cell].

### Morphological analysis of the hair cell pairs during inversion

Circularity of the pair was calculated for all timeframes at which the nascent hair cells shared a bond, as:

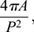
where *A* is the sum of the area of both cells and *P* is the sum of the perimeter of both cells minus two times the length of their shared membrane segment. To determine the interfacial shape, the lines corresponding to the membrane interface between rotating pairs were exported as a list of *xy* coordinates and rotated and centered such that both ends laid on the 0 of the *y*-axis and equidistant to the 0 on the *x*-axis. A S or Ƨ shape was assigned depending on the asymmetry of the line on the *x*-axis. The line was classified as a C shape depending on its asymmetry on the *y*-axis, if it was not previously classified as S or Ƨ. If the interface had no notable asymmetry on either axis, it was classified as straight.

### Analysis of final hair cell angle

The final angle for the three experimental conditions is defined as the angle of the vector that goes through both hair cell sibling cells with respect to the horizontal axis. Given the cartesian coordinates of each pair 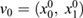 and 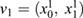, the vector *v*_*f*_=*v*_0_−*v*_1_ connects both cells. As, in principle, any of the two cells can be labeled as zero or one, the orientation of the vector is not important for the calculation. Thus, the angle is calculated as:

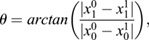
which appropriately gives all angles in the first quadrant.

Then, the angle distributions were compared by pairs using a Kolmogorov-Smirnov test to evaluate whether the two samples belong to the same distribution. This was performed using SciPy's statistical package (Virtanen et al., 2020).

### Computational model

In order to better understand how the *in vivo* rotations progress and stop, we decided to develop a mathematical and computational model. We chose a Monte Carlo Metropolis minimization scheme as it combines deterministic potentials to model the stop and stochastic evolution for the progression. In our model, each of the two cells is characterized by a particle in a one-dimensional space, representing the angle with respect to the A-P axis. Thus, the positions *θ*=0 and *θ*=180° correspond to opposing sides of that anatomical axis. The anterior region is defined as the second and third quadrant, while the posterior region is the first and fourth one (Fig. 7A). Each cell is subject to certain potentials. Cells see one another through a soft sphere repulsion, taken from the repulsive term of a Lennard-Jones potential.

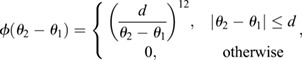
where *d* is the maximum interaction distance and *θ*_1_ and *θ*_2_ are the positions of both cells. As the hair cell pair is bound throughout the rotation at all times in the *in vivo* experiments, parameter *d* is chosen as 180° to account for an interaction in the whole domain.

We also assumed that each cell is ruled by an attractor in the form of a Gaussian well,

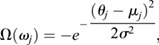
where *θ*_*j*_ is the position of cell *j*, *μ*_*j*_ is the position of the attractor's minima for cell *j* and *σ* is the attractor's standard deviation. Both potentials are encoded in the system's energy, through a Hamiltonian, as:

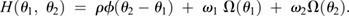


The final configuration of the system is reached through an evolution, according to a Metropolis-Hasting algorithm for the phase space sampling (Metropolis et al., 1953; Hastings, 1970). The coefficients *ρ*, *ω*_1_ and *ω*_2_ allow the control of each potential's strength, representing the repulsion strength and the depth of each well, respectively. After an initial breaking time, the potential is turned on by setting *ω*_1_, *ω*_2_ or both to non zero values. The initial positions of the cells in the simulation are taken from a normal distribution. Its parameters are obtained through a fit of a Gaussian function to the experimental initial positions of the hair cells pairs, just after the progenitor's division, choosing *μ*_*initial*_=180^°^±50^°^ as an initial condition. The one-dimensional space is arbitrarily divided in a 0.25-degree interval, which is the smallest possible position change. Thereafter, cells may update their position as an anti-clockwise or clockwise movement, one cell at a time. This movement is represented by normalized velocities +1 and −1 in the discreet space, respectively. The change in energy due to the new position is then compared with the previous energy state. If the system's energy is reduced, the change is accepted with a probability of 1, otherwise it is accepted with a probability drawn from a Boltzmann distribution. When the change is rejected, the corresponding cell does not move. These calculations are performed for a fixed number of time steps for all simulations.

## Supplementary Material

10.1242/develop.200975_sup1 Supplementary information
