# Supplementary material for: Quantitative videomicroscopy reveals latent control of cell-pair rotations in vivo
Source: Development. 2023 May 3;150(9):dev200975. doi: 10.1242/dev.200975 (PMC10259515; doi:10.1242/dev.200975)
Supplement: Supplementary information [file develop-150-200975-s1.pdf]

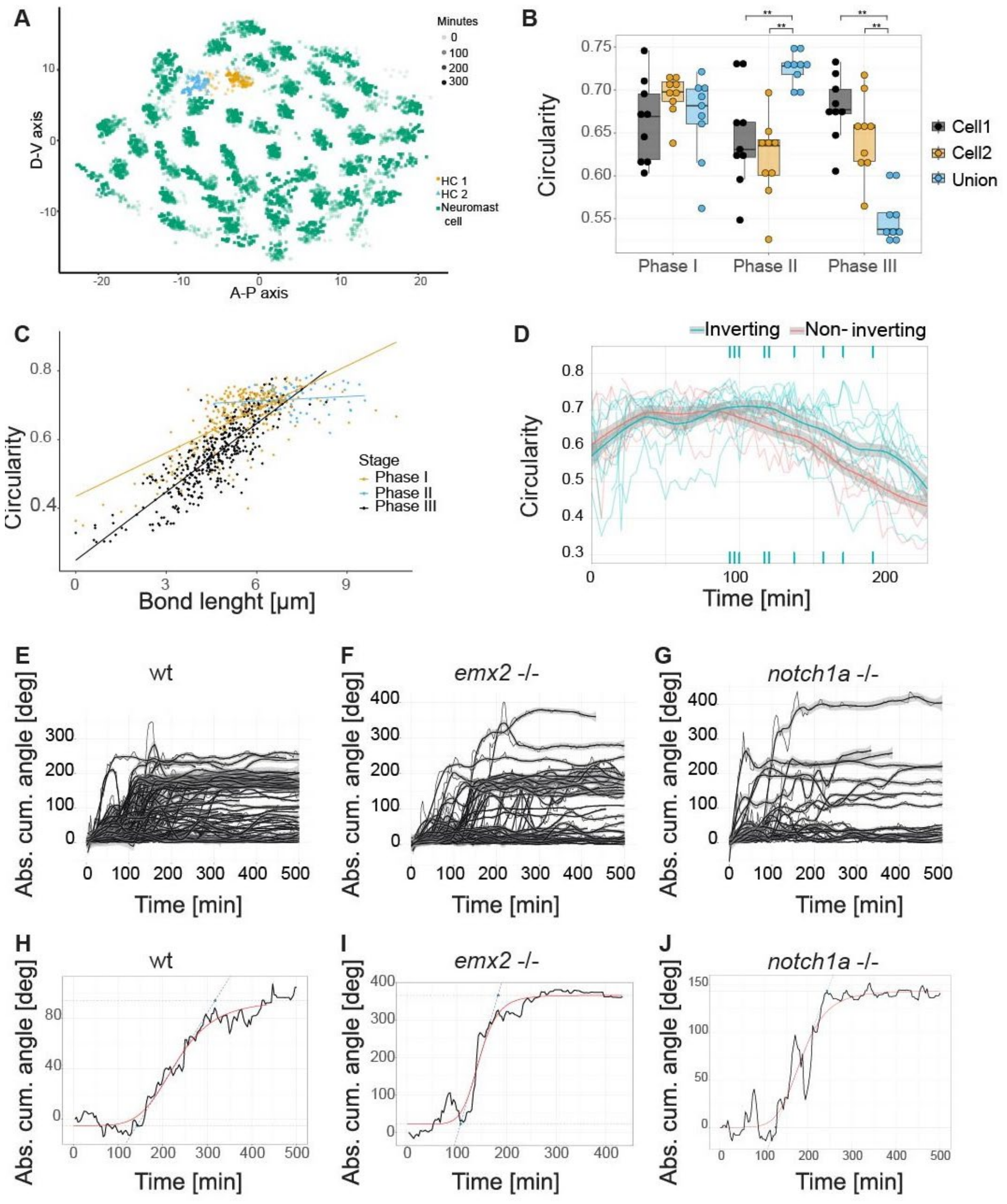

Fig. S1. (A) Epithelium-wide cell tracking during a hair cell pair re-arrangement. Each point marks the centroid position of a neuromast cell. Minute 0 marks the time when a hair cell progenitor divides to give rise to HC1 (Yellow) and HC2 (Cyan) (B) Comparing circularity of individual cells of the inverting pair and their union during different phases. During Phase 1, circularities of cell1, cell2 and the union of both are similar. Both cells deform in a correlated manner during Phase 2 so that their union is significantly more circular than either one separate. Within-phase comparisons were done with a Wilcox test, significant

Development • Supplementary information

differences with a p value < 0.01 are marked with an **. (C) Correlation of cell pair circularity and bond length. Each point represents a timepoint from any of 9 different inverting cell pairs. Throughout the whole process and experiments, the correlation between both observables is high (0.79 spearman correlation). Phase 2 shows the highest circularity and bond length while Phase 3 depicts the lowest on both magnitudes. (D) Circularity over time for sibling cells, beginning right after their birth. The LOESS smoothing (locally weighted smoothing) is shown for the inverting (blue) and non-inverting (red) cell pairs. The blue ticks at the top and bottom indicate the time of inversion of individual pairs. (E-G) Absolute cumulative angle vs. time of cell pairs from wt (E), emx2 mutant (F) and notch1a mutant (G) larvae. Included are non-inverting pairs (the trajectories that remain close to 0 degrees) as well as inverting pairs (the trajectories that rise and remain close to 180 degrees). Note that only a few cell pairs rotate more than 180 degrees (full inversion). (H-J) Examples of cell-pair inversion process and how the inversion timings were determined for the inverting cell pairs from wt (H), emx2 mutant (I) and notch1a mutant (J) larvae. The black solid lines are the measured angular data through time, while the red line marks the logistic fit. A diagonal dotted line marks the linear regime of the logistic function; where it crosses the lower and upper asymptotes it defines, respectively, the onset and termination of inversions.

Development • Supplementary information

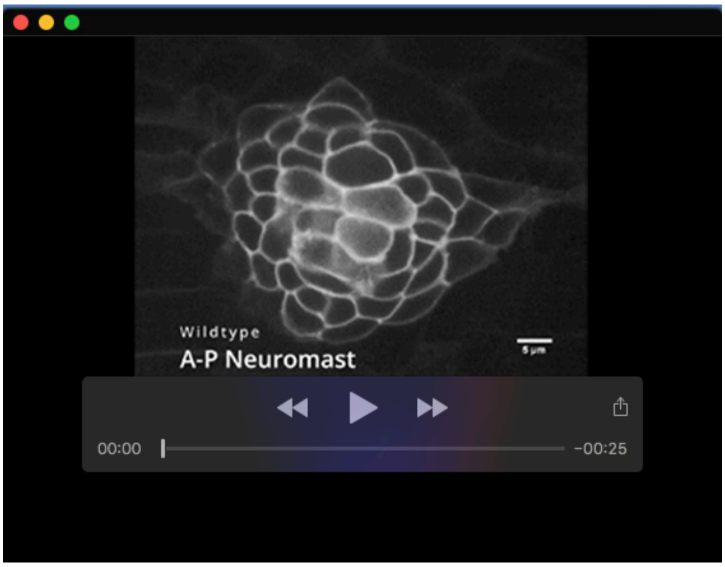

Movie 1. A wild-type horizontal neuromast with a focus on a dividing UHCP that results in a pair of nascent hair cells inverting. Membranes are marked by the claudnb:lyn-EGFP transgenic. Hair cells are marked simultaneously by myo6b:β-actin-GFP that also allows us to see the orientation of their hair bundle.

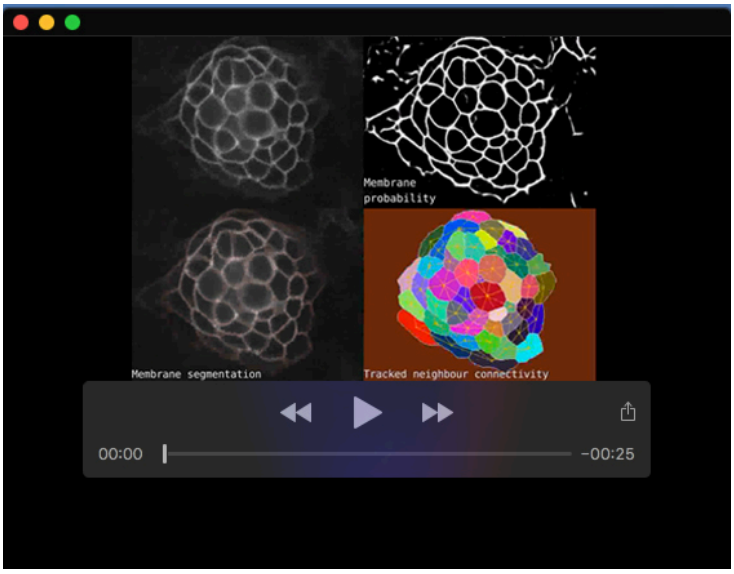

Movie 2. Illustration of key steps in the segmentation and tracking of cell-pair rotation timelapses. Membrane probability: output of the Ilastik autocontext workflow trained to classify pixels as membrane/non-membrane. The probability is an input for the Ilastik multicut workflow and the resulting skeletonized segmentation of cell boundaries is manually refined in Tissue analyser. Cells are then tracked over time to give an evolving topological representation of the tissue in general, and the cell-pair rotation in particular.

Development • Supplementary information

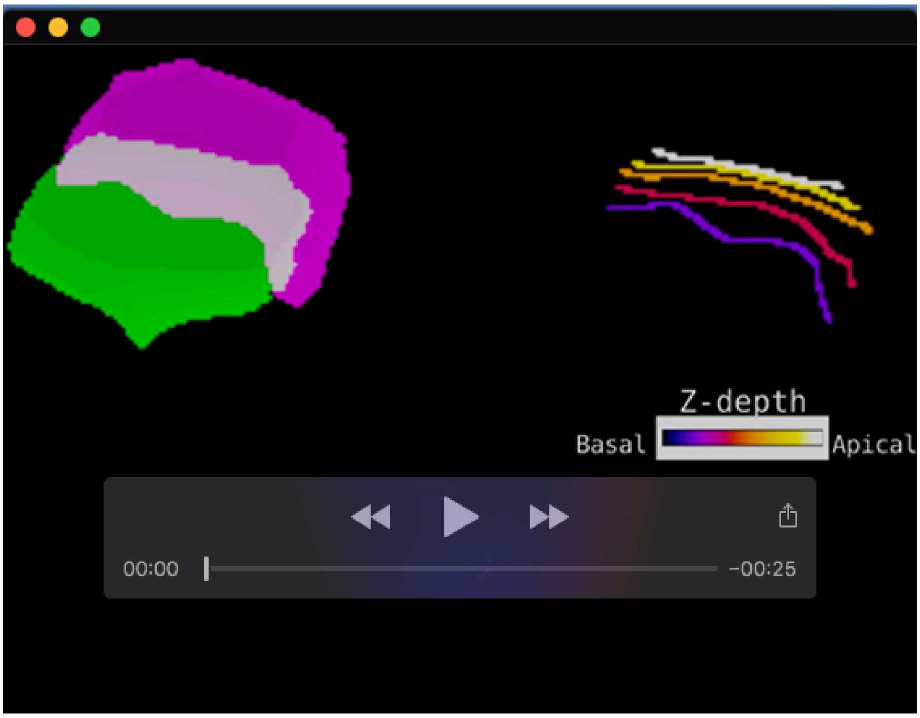

Movie 3. A subset of the inverting pairs were segmented over 4 to 5 Z-slices with 1 μm apart. The membrane segments separating the siblings

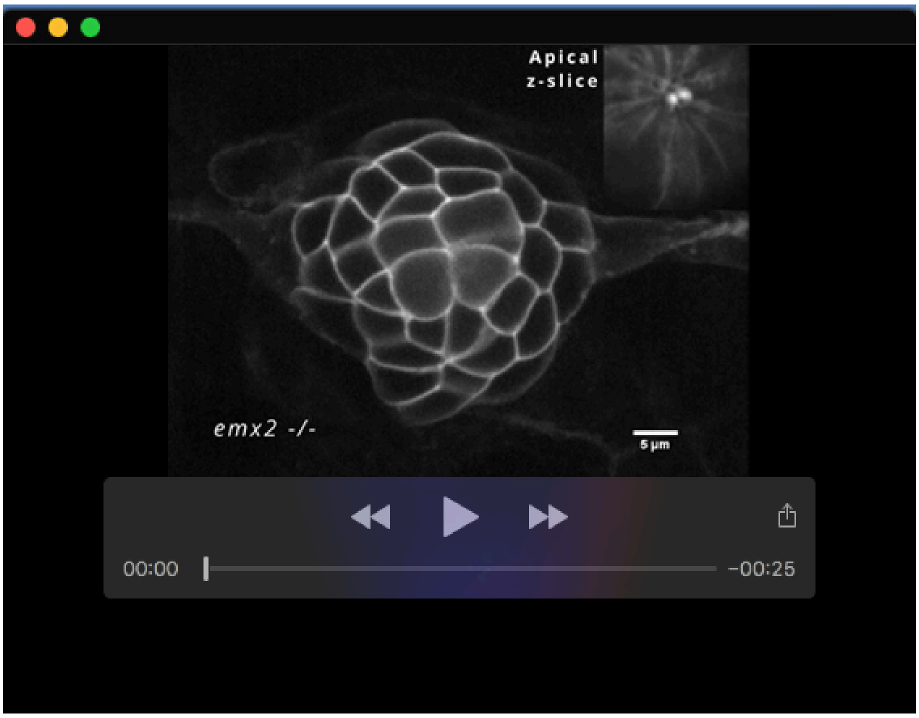

Movie 4. A horizontal neuromast of *emx2* mutant larva showcasing an inverting pair of hair cells. Their anteriorly positioned centrioles can be seen towards the end of the movie.

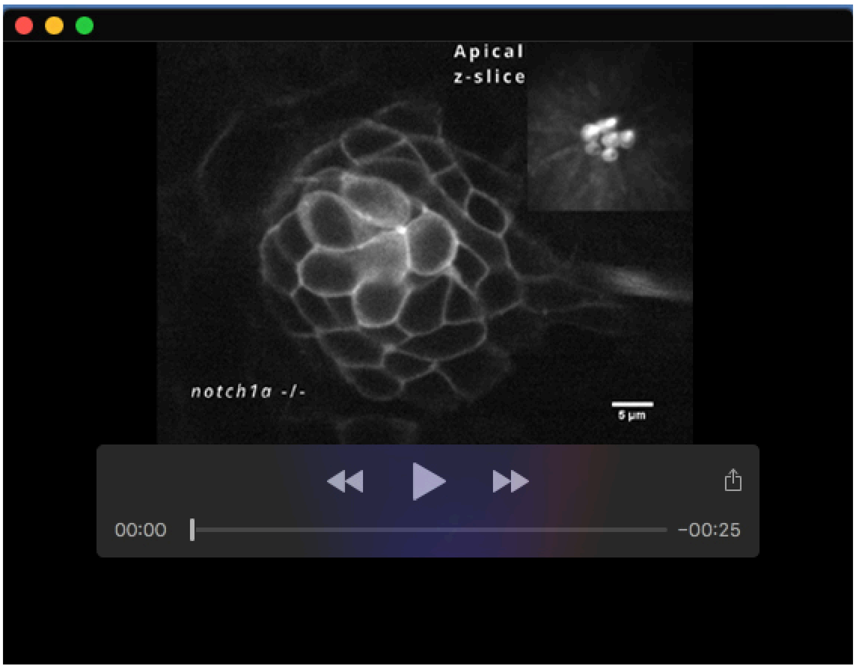

Movie 5. A horizontal neuromast of *notch1a* mutant larva in which a UHCP divides to give rise to a rotating pair of hair cells. The orientation of both cells' hair bundles is towards the posterior.

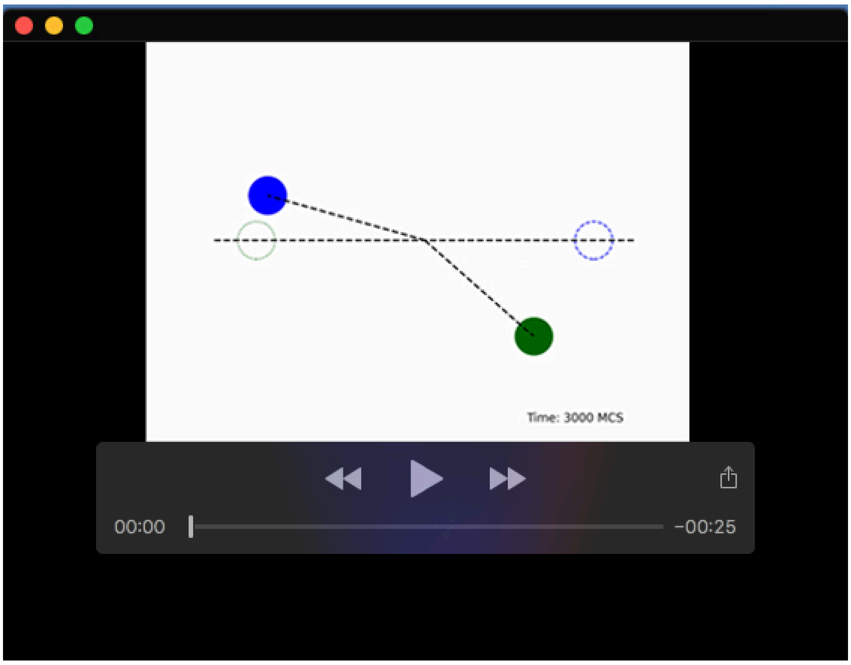

Movie 6. Asymmetrical model dynamic: Until a symmetry breaking event at 10000 MCS, each cell, represented by solid circles, has their respective gaussian well on sites opposite to their starting position in the AP axis, drawn as a dotted circle of a matching color. The strength of the attraction is represented by the size of the dashed circle. The angle with respect to the AP axis is defined as the angle between the AP axis and line that joins the two cells.

Development • Supplementary information

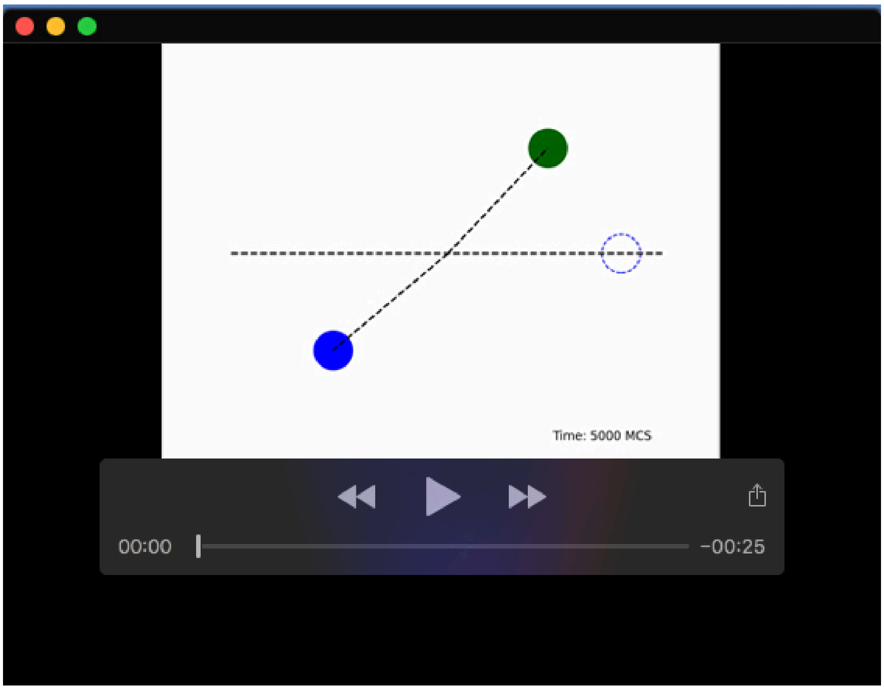

Movie 7. One well model dynamic: Only the blue cell has a gaussian well (dashed blue circle) on the site opposite to their initial position in the AP axis. After 10000 Monte Carlo steps, the attraction well is turned on. The green cell has an attraction well but its attraction force is set to 0. The green cell is pushed by the repulsion of the blue one.

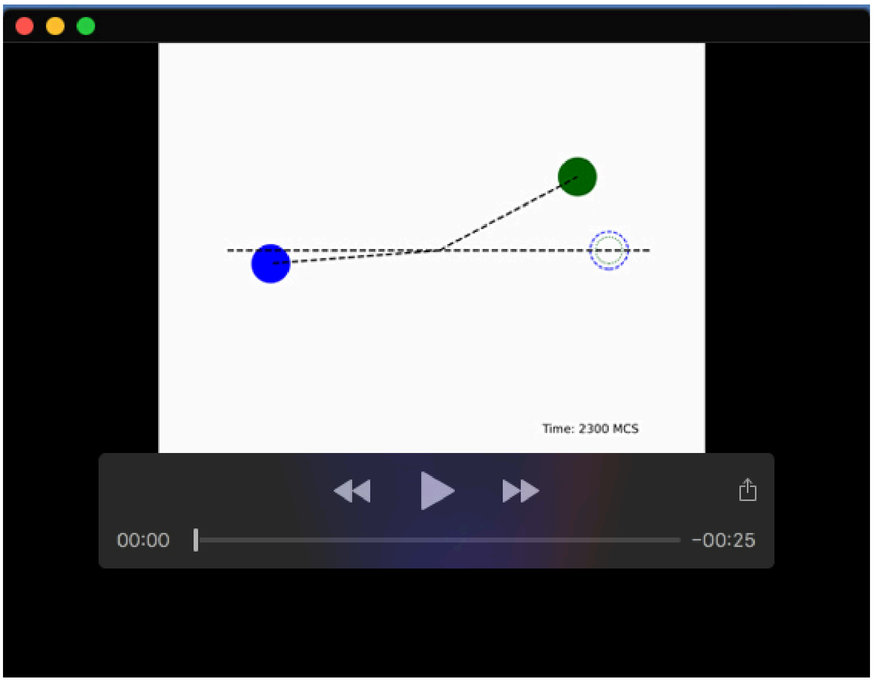

Movie 8. Symmetrical model dynamic: Until a symmetry breaking event at 10000 MCS, each cell, represented by solid circles, has their respective gaussian well on the same side of the AP axis, drawn as a dotted circle of a matching color, while the strength of the attraction is represented by the size of the dashed circle. The angle with respect to the AP axis is defined as the angle between the AP axis and line that joins the two cells.
