# Supplementary material for: Quantitative videomicroscopy reveals latent control of cell-pair rotations in vivo
Source: Development. 2023 May 3;150(9):dev200975. doi: 10.1242/dev.200975 (PMC10259515; doi:10.1242/dev.200975)
Supplement: Supplementary file 1 [file develop-150-200975_review_history.pdf]

Quantitative videomicroscopy reveals latent control of cell-pair rotations *in vivo*

Eva L. Kozak, Jerónimo R. Miranda-Rodríguez, Augusto Borges, Kai Dierkes, Alessandro Mineo, Filipe Pinto-Teixeira, Oriol Viader-Llargués, Jérôme Solon, Osvaldo Chara and Hernán López-Schier

DOI: 10.1242/dev.200975

Editor: Steve Wilson

Review timeline

Original submission:	6 June 2022
Editorial decision:	12 July 2022
First revision received:	25 January 2023
Accepted:	20 February 2023

Original submission

First decision letter

MS ID#: DEVELOP/2022/200975

MS TITLE: Quantitative videomicroscopy reveals latent control of cell-pair rotations in vivo

AUTHORS: Hernan Lopez-Schier, Osvaldo Chara, Eva L Kozak, Jeronimo R Miranda-Rodriguez, Augusto Borges, Kai Dierkes, Alessandro Mineo, Filipe Pinto-Teixeira, Oriol Viader-Llargues, and Jerome Solon

I have now received all the referees' reports on the above manuscript, and have reached a decision. The referees' comments are appended below, or you can access them online: please go to BenchPress and click on the 'Manuscripts with Decisions' queue in the Author Area.

As you will see, the referees express considerable interest in your work, but have some criticisms and suggestions for improving your manuscript. If you are able to revise the manuscript along the lines suggested, I will be happy receive a revised version of the manuscript. If it would be helpful, you are welcome to contact us to discuss your revision in greater detail.

Please attend to all of the reviewers' comments and ensure that you clearly highlight all changes made in the revised manuscript. Please avoid using 'Tracked changes' in Word files as these are lost in PDF conversion. I should be grateful if you would also provide a point-by-point response detailing how you have dealt with the points raised by the reviewers in the 'Response to Reviewers' box. If you do not agree with any of their criticisms or suggestions please explain clearly why this is so.

Reviewer 1

Advance summary and potential significance to field

In their manuscript, Kozak and colleagues report a quantitative description of the rotation of the pairs of hair cells in the neuromasts of zebrafish embryos. The hair cells of the neuromasts are sensing the flow of water outside the embryo and are the sensory organs of the lateral line organ in fish (and amphibians). Using imaging, the authors collect high resolution (spatial and temporal)

© 2023. Published by The Company of Biologists under the terms of the Creative Commons Attribution License (https://creativecommons.org/licenses/by/4.0/).

time lapse movies of rotating hair cells in wild-type, notch1a, and emx2 mutants and use different ways to analyze the rotatory movement. From these movies/analyses, the authors conclude several things. Frist, hair cell pair rotation is an active, likely autonomous process. Second, the hair cell pair maintains cell-cell contact but remodels its contacts to the neighboring cells during rotation. Third, hair cell pair rotations are (mostly) independent of notch1a and emx2 activity, suggesting that the molecular asymmetry between the two hair cells forming a hair cell pair does not influence rotation. Fourth, notch1a and emx2 activity are required to reduce the noisiness in hair cell pair rotation. Together, this description provides a quantitative understanding of hair cell rotation in neuromasts and by extension also in other instances where cell pairs rotate.

Comments for the author

This study is carefully conducted and well presented. The movies are of excellent quality, statistics are reported, and the conclusions are supported by the measurements. My main “concern” is that this study is descriptive and offers little insight into how the hair cell pair rotates (do the cells actually crawl in a circle or do they rearrange/remodel their junctions to change positions? What is the stop signal for rotation?).

Minor:
1. Fig. 2 “Strong homotypic cell-cell interactions” – what is the evidence for homotypic interactions and strong interactions?
2. Density plots – not sure how the density profile was obtained but maybe binning might be better than a continuous plot for discreet data? eg. Fig. 1F and the like.

<u>Reviewer 2</u>

Advance summary and potential significance to field

In this paper, Kozak et al. make use of the zebrafish lateral line to study cell rotation, whereby two cells swap position through coordinated rotation, in organogenesis. Studying this type of cell behaviour is interesting because it allows for establishing candidate mechanisms and pathways that promote symmetry breaking in cells. The present work offers a detailed, quantitative, and sophisticated study of sibling hair cell rotation in lateral line development. The most important findings from this work are that sibling hair cell rotation i) depends on both cells contributing to a pair, and ii) does not require Notch1a activity, which challenges previous work from another group. Moreover, capitalising on their new quantitative method, the authors present results that suggest a role for Notch1(and Exm2) in limiting wobbling of the cell pair during rotation which the authors interpret as Notch1a limiting noise during this process.

Comments for the author

Overall, this study examines an interesting process in morphogenesis. It is primarily descriptive, which is of course fine, as the author’s methodology is very rigorous and will be useful for future studies. However, the manuscript perhaps lacks in mechanistic insight. It would be advantageous to understand the wobbling phenotype. Does it result from altered ActomyosinII activity/regulation? Changes in cell adhesion? other candidate pathways, potentially explaining Notch1a function in "precision".
I feel that examining these possibilities should be feasible and would make the study of interest to a broader audience of cell and developmental biologists.

Minor comments
- I would consider trying to simplify the paper, which at time feels a bit dense in terms of background information (result section).
- “…/… cells neither push, nor pull one another during rotations (Brangwynne et al., 2000). However subsequent theoretical analyses concluded that the cells must employ rear pull during rotations (Camley et al., 2014; Leong, 2013) …/…” Should be “previous”

© 2023. Published by The Company of Biologists under the terms of the Creative Commons Attribution License (https://creativecommons.org/licenses/by/4.0/).

First revision

Author response to reviewers' comments

Point-by-point explanation to reviewers

Reviewer 1 Advance summary and potential significance to field
In their manuscript, Kozak and colleagues report a quantitative description of the rotation of the pairs of hair cells in the neuromasts of zebrafish embryos. The hair cells of the neuromasts are sensing the flow of water outside the embryo and are the sensory organs of the lateral line organ in fish (and amphibians). Using imaging, the authors collect high resolution (spatial and temporal) time lapse movies of rotating hair cells in wild-type, notch1a, and emx2 mutants and use different ways to analyze the rotatory movement. From these movies/analyses, the authors conclude several things. Frist, hair cell pair rotation is an active, likely autonomous process. Second, the hair cell pair maintains cell-cell contact but remodels its contacts to the neighboring cells during rotation. Third, hair cell pair rotations are (mostly) independent of notch1a and emx2 activity, suggesting that the molecular asymmetry between the two hair cells forming a hair cell pair does not influence rotation. Fourth, notch1a and emx2 activity are required to reduce the noisiness in hair cell pair rotation. Together, this description provides a quantitative understanding of hair cell rotation in neuromasts and by extension also in other instances where cell pairs rotate.

Comments for the authorThis study is carefully conducted and well presented. The movies are of excellent quality, statistics are reported, and the conclusions are supported by the measurements. My main “concern” is that this study is descriptive and offers little insight into how the hair cell pair rotates (do the cells actually crawl in a circle or do they rearrange/remodel their junctions to change positions? What is the stop signal for rotation?).

We thank the reviewer for praising the overall quality of the work. We believe that the original manuscript did offer a mechanistic glimpse. Yet, the new version that includes a computer simulation of the process, gives a much more profound insight of the mechanism underlying rotations. The question of “What is the stop signal for rotation?” is very important because it zeroes in on key differences between all previous in vitro and in silico studies and the in vivo scenario that we have tackled. Cells in vitro rotate indefinitely. However, in vivo rotations occur just once. Moreover, the final angle of cells once rotations end is tightly controlled. This difference is very important because it highlights two aspects of rotations in vivo which could not have been predicted from studies in vitro. One is the putative role of surrounding cells in constraining rotations. The second is the existence of a Cartesian coordinate system that informs cells about orthogonal axes of the neuromast epithelium. We addressed this issue by developing a new computational model. This model, as it recapitulates the essential characteristics of the process under study, establishes the minimal requirements for the cellular rearrangements in vivo, and provides a likely answer to the question of “What is the stop signal for rotation?. Different variants of a two-cell system, which assume that each cell is attracted to an energy "well" where its potential energy is minimal, and that each cell will also be subject to repulsion due to the space exclusion of the other cell, recapitulate the three phase angular movement observed in experiments. We would like to highlight that the overall kinetics of the angle between the two cells, the final resting angle, as well as the rotational noise predicted by the computational model capture with remarkable accuracy the empirical results. In short, we conclude that directional asymmetry links rotational and positional precision, explained in a new Figure 7. Of note, the previous Figure 7 “A model of cell-pair rotation in vivo” is now Figure 8.

We also developed a model variant in which the attraction wells are not located on opposite sides of the anteroposterior axis of the epithelium, but rather are on top of each other. As a result, cells compete to reach the same position. This model explains the dynamics of cells upon loss of Notch1a (which makes both cells indistinguishable with respect to Emx2 expression) and, conversely, also the dynamics from the loss of Emx2. In both cases the final angle of the cell pair is not as well aligned to the anteroposterior axis of the epithelium despite the fact that the attraction wells remain invariably positioned. Therefore, starting from basic assumptions, we demonstrate that the experimental characteristic of rotations - the stopping and the wobbling- can be linked to the transcriptional asymmetry between sibling cells. Although a significant amount remains to be

© 2023. Published by The Company of Biologists under the terms of the Creative Commons Attribution License (https://creativecommons.org/licenses/by/4.0/).

learned, our work offers new mechanistic insight by showing that the loss of symmetry breaking before rotations is sufficient to explain all the observed phenotypes at a quantitative level.

Minor:
1. Fig. 2 “Strong homotypic cell-cell interactions” – what is the evidence for homotypic interactions and strong interactions?

We used “Strong homotypic cell-cell interactions” to refer to the contact between the nascent hair cells as well as between the rotating cell pair and their neighbors, respectively homotypic and heterotypic. The homotypic contact is conserved throughout the process (the pair does not separate until much later) and even grows in length and is maximum at the time of rotation (Fig. 2E). However, to avoid confusion, we changed the word “interactions” to “contacts” when referring to the cell-cell membrane contacts.

2. Density plots – not sure how the density profile was obtained but maybe binning might be better than a continuous plot for discrete data? eg. Fig. 1F and the like.

We believe that density plots offer the clearest visualization as we present more than one distribution simultaneously and, therefore, a continuous density line is easier to follow and avoid overlaps. The number of points in our dataset justify the use of a continuous approximation for the graphs.

Reviewer 2 Advance summary and potential significance to field In this paper, Kozak et al. make use of the zebrafish lateral line to study cell rotation, whereby two cells swap position through coordinated rotation, in organogenesis. Studying this type of cell behaviour is interesting because it allows for establishing candidate mechanisms and pathways that promote symmetry breaking in cells. The present work offers a detailed, quantitative, and sophisticated study of sibling hair cell rotation in lateral line development. The most important findings from this work are that sibling hair cell rotation i) depends on both cells contributing to a pair, and ii) does not require Notch1a activity, which challenges previous work from another group. Moreover, capitalising on their new quantitative method, the authors present results that suggest a role for Notch1(and Exm2) in limiting wobbling of the cell pair during rotation, which the authors interpret as Notch1a limiting noise during this process.

Comments for the authorReviewer 2 Comments for the author
Overall, this study examines an interesting process in morphogenesis. It is primarily descriptive, which is of course fine, as the author’s methodology is very rigorous and will be useful for future studies. However, the manuscript perhaps lacks in mechanistic insight. It would be advantageous to understand the wobbling phenotype. Does it result from altered ActomyosinII activity/regulation? Changes in cell adhesion? other
candidate pathways, potentially explaining Notch1a function in "precision".
I feel that examining these possibilities should be feasible and would make the study of interest to a broader audience of cell and developmental biologists.

This is an important question. We never detected any differences indicating asymmetries in membrane tension or cell adhesion. Specifically, (1) We do not see any consistent asymmetry in the rate of neighbor exchanges. When we find asymmetries, they fluctuate and are not correlated with the Emx2/Notch1a status of the cells (Fig. 3C,D). This suggests that their Emx2/Notch1a status is not determinant of the adhesion of the rotating cells to its neighbors.
(2) If there were significant differences in actomyosin tension between the Notch1a+ and the Emx2+ siblings, we would expect that the cell with the lowest tension would show signs of “engulfment” of the cell with the highest membrane tension during rotation, resulting in a consistent and conspicuous “C” shape interface. We seldomly saw this shape. Instead, the most commonly seen interface was a straight line, indicating that a sustained difference in surface tension between the siblings is very unlikely (Fig. 3A,B).

In addition to the experimental data, the computational model argues that wobbling can be explained by a loss of Notch/Emx2 asymmetry and both cells competing to reach the same position. The model suggests that the same mechanism simultaneously drives: (1) a precise end of the rotations and (2) the smoothness of the movement (See answer to the reviewer 1). Importantly,

© 2023. Published by The Company of Biologists under the terms of the Creative Commons Attribution License (https://creativecommons.org/licenses/by/4.0/).

What ties both processes is very likely Notch/Emx2 intercellular asymmetry. Regrettably, currently the field has neither provided, nor persuasively argued, for the exact molecular identity of transcriptional asymmetries at the level or downstream Notch/Emx2, precluding more detailed tests. This point is now presented in a new section entitled “Computational modeling of the inversion process suggests that directional asymmetry is the link between rotational and positional precision”. A new Figure 7 describes the computer simulations. Of note, the previous Figure 7 “A model of cell-pair rotation in vivo” is now Figure 8.

Minor comments
- I would consider trying to simplify the paper, which at time feels a bit dense in terms of background information (result section).

According to this suggestion, some paragraphs were moved from the results section to the methods section or to the discussion.
- The paragraph:
“The arc-length should not be confused with the closely related “turn” (the absolute cumulative angle). For the turn, we summed the angular changes taking into account their sign and then took the absolute value, whereas for the arc-length we took the absolute of angular changes at each step and then summed them all. In short, the turn is the angular position relative to the initial position, whereas the arc-length is the sum of the total angular distance traveled in any direction.” Was deleted from the results section as this is already described in the methods section “comparison of cell-pair inversion process in distinct genotypes”

-The sentence “In the videographs, newborn hair cells were identified retrospectively by playing the movies backwards from the time when hair cells can be unambiguously defined using validated transgenic markers (López-Schier and Hudspeth, 2006; Kindt et al., 2012).”
Was moved to the methods section.

- “…/… cells neither push, nor pull one another during rotations (Brangwynne et al., 2000). However, subsequent theoretical analyses concluded that the cells must employ rear pull during rotations (Camley et al., 2014; Leong, 2013) …/…” Should be “previous”

We thank the reviewer for pointing this out. We have changed the wording to make it more precise. “However, theoretical analyses concluded that the cells must employ rear pull during rotations (Camley et al., 2014; Leong, 2013).”

Second decision letter

MS ID#: DEVELOP/2022/200975

MS TITLE: Quantitative videomicroscopy reveals latent control of cell-pair rotations in vivo

AUTHORS: Hernan Lopez-Schier, Osvaldo Chara, Eva L Kozak, Jeronimo R Miranda-Rodriguez, Augusto Borges, Kai Dierkes, Alessandro Mineo, Filipe Pinto-Teixeira, Oriol Viader-Llargues, and Jerome Solon
ARTICLE TYPE: Research Article

I am happy to tell you that the reviewers are happy with your revisions and your manuscript has been accepted for publication in Development, pending our standard ethics checks. The referee reports are appended below.

Reviewer 1

Advance summary and potential significance to field

This study provides a quantitative insight into cell pair rotations. Although it lacks mechanistic insight, the modeling of the rotation of a cell pair provides an entry point to tackle this issue in future studies.

© 2023. Published by The Company of Biologists under the terms of the Creative Commons Attribution License (https://creativecommons.org/licenses/by/4.0/).

Comments for the author

This is a beautiful study and the revisions made it even nicer. My slight concern about mechanistic insight was somewhat addressed using a modeling approach - although this provides merely an idea of how the cell pair might be rotating. Identification of the nature of the attraction (the gaussian wells in the mdoel) would provide a real mechanistic insight. But this is hard and beyond the scope of this study I feel.

Reviewer 2

Advance summary and potential significance to field

The authors have addressed my comments and i think the revised version of the manuscript will be of broad interest to the community of cell and developmental biologists.

Comments for the author

The authors have addressed my comments and i think the revised version of the manuscript will be of brand interest to the community of cell and developmental biologists.

© 2023. Published by The Company of Biologists under the terms of the Creative Commons Attribution License (https://creativecommons.org/licenses/by/4.0/).
